# Baseline colitogenicity and acute perturbations of gut microbiota in immunotherapy-related colitis

**DOI:** 10.1084/jem.20232079

**Published:** 2024-12-12

**Authors:** Joan Shang, Diane Marie Del Valle, Graham J. Britton, K.R. Mead, Urvija Rajpal, Alice Chen-Liaw, Ilaria Mogno, Zhihua Li, Rajita Menon, Edgar Gonzalez-Kozlova, Arielle Elkrief, Jonathan U. Peled, Tina Ruth Gonsalves, Neil J. Shah, Michael Postow, Jean-Frederic Colombel, Sacha Gnjatic, David M. Faleck, Jeremiah J. Faith

**Affiliations:** 1 Precision Immunology Institute, Icahn School of Medicine at Mount Sinai, New York, NY, USA; 2 Icahn Institute for Data Science and Genomic Technology, Icahn School of Medicine at Mount Sinai, New York, NY, USA; 3 Human Immune Monitoring Center, Icahn School of Medicine at Mount Sinai, New York, NY, USA; 4 Tisch Cancer Institute, Icahn School of Medicine at Mount Sinai, New York, NY, USA; 5 Oncological Sciences, Icahn School of Medicine at Mount Sinai, New York, NY, USA; 6 Vedanta Biosciences, Cambridge, MA, USA; 7 Department of Medicine, Memorial Sloan Kettering Cancer Center, New York, NY, USA; 8 Human Oncology and Pathogenesis Program, Memorial Sloan Kettering Cancer Center, New York, NY, USA; 9 Department of Pathology, Memorial Sloan Kettering Cancer Center, New York, NY, USA; 10 Department of Medicine, Adult Bone Marrow Transplantation Service, Memorial Sloan Kettering Cancer Center, New York, NY, USA; 11 Weill Cornell Medical College, New York, NY, USA; 12 Division of Gastroenterology, Icahn School of Medicine at Mount Sinai, New York, NY, USA; 13 Department of Medicine, Icahn School of Medicine at Mount Sinai, New York, NY, USA; 14 Pathology, Molecular and Cell-Based Medicine, Icahn School of Medicine at Mount Sinai, New York, NY, USA

## Abstract

Immunotherapy-related colitis (irC) frequently emerges as an immune-related adverse event during immune checkpoint inhibitor therapy and is presumably influenced by the gut microbiota. We longitudinally studied microbiomes from 38 ICI-treated cancer patients. We compared 13 ICI-treated subjects who developed irC against 25 ICI-treated subjects who remained irC-free, along with a validation cohort. Leveraging a preclinical mouse model, predisease stools from irC subjects induced greater colitigenicity upon transfer to mice. The microbiota during the first 10 days of irC closely resembled inflammatory bowel disease microbiomes, with reduced diversity, increased Proteobacteria and *Veillonella*, and decreased *Faecalibacterium*, which normalized before irC remission. These findings highlight the irC gut microbiota as functionally distinct but phylogenetically similar to non-irC and healthy microbiomes, with the exception of an acute, transient disruption early in irC. We underscore the significance of longitudinal microbiome profiling in developing clinical avenues to detect, monitor, and mitigate irC in ICI therapy cancer patients.

## Teaser

Pretreatment gut microbiomes of immunotherapy-related colitis patients drive worse colitis in susceptible mice compared with non-colitis cancer immunotherapy microbiomes. Microbiome shifts in immunotherapy-related colitis initially mirror inflammatory bowel disease patients but subsequently resolve suggesting a reversible, colitogenic state.

## Introduction

The intestinal microbiome is an essential contributor to host health, which includes the development and homeostatic maintenance of healthy barrier tissue and the immune system (Hooper and Macpherson, 2010). Changes in the gut microbiota, including reduced microbiota biodiversity, reduced density, loss of beneficial commensals, or expansion of pathobionts, have been implicated in a wide array of diseases, including cancer and inflammatory conditions such as inflammatory bowel disease (IBD) (Routy et al., 2018; Matson et al., 2018; Frankel et al., 2017; Sivan et al., 2015; Lee et al., 2022; Spencer et al., 2021; Derosa et al., 2020; Frank et al., 2007; Baruch et al., 2021; Andrews et al., 2021; Davar et al., 2021; Chaput et al., 2017; Simpson et al., 2022; McCulloch et al., 2022; Arthur et al., 2012). Studies have identified bacteria that are associated with clinical response to immune checkpoint inhibitors (ICI), and antibiotics are negatively associated with ICI response (Matson et al., 2018; Frankel et al., 2017; Sivan et al., 2015; Spencer et al., 2021; Derosa et al., 2020; Baruch et al., 2021; Andrews et al., 2021; Davar et al., 2021; McCulloch et al., 2022; Fidelle et al., 2023; Routy et al., 2018, 2023; Elkrief et al., 2023; Vétizou et al., 2015; Shaikh et al., 2021). Recent studies also suggest that the microbiota may influence the development of immune-related adverse events (irAEs) in ICI-treated patients (Hooper and Macpherson, 2010; Chaput et al., 2017; Frankel et al., 2017; Simpson et al., 2022; Wang et al., 2018b; Dubin et al., 2016; Elkrief et al., 2023; Wang et al., 2020; Halsey et al., 2023). Immunotherapy-related colitis (irC) can pose life-threatening risks and is among the most common irAE associated with anti-CTLA-4 and dual anti-PD-1/anti-CTLA-4 (Som et al., 2019; Khoja et al., 2017). Gut microbiotas of patients with gastrointestinal (GI)-related irAEs are distinct from patients with non-GI irAEs (Liu et al., 2019), and recent studies have suggested that a subject’s baseline (before ICI therapy was started) microbiota can predict irC risk (Lee et al., 2022; Andrews et al., 2021; Chaput et al., 2017; Wang et al., 2018b; Dubin et al., 2016; Liu et al., 2019; Abu-Sbeih and Wang, 2020). However, the microbiome’s contribution to irC remains elusive, as we do not know the timing of these microbiome changes, if the baseline microbiome has a causal role in disease, or if there are parallels between the microbiome’s role in irC and IBD. Fecal microbiota transplantation (FMT) of healthy donor stool to patients with treatment-refractory irC led to improved symptoms or complete resolution in 17 out of 19 patients across existing non-placebo-controlled small trials (Wang et al., 2018b; Elkrief et al., 2023; Wang et al., 2020; Halsey et al., 2023), suggesting a potential causal role for the gut microbiota in irC.

Longitudinal gut microbiome sampling prior to disease onset, during active disease, and after disease resolution is one of the most sought-after resources to understand the gut microbiome’s role in the etiology of immune-mediated conditions. However, microbiome sampling predisease is rarely conducted due to intense resource and logistical challenges, limiting these studies to large multisite efforts for IBD (Raygoza Garay et al., 2023) and Type 1 diabetes (Stewart et al., 2018). The rapid onset of irC in patients under anti-CTLA-4 and dual-ICI treatment provides an opportunity to expand our understanding of the gut microbiome’s role in immune-mediated conditions from predisease to disease resolution.

We present an in-depth characterization of the microbiota dynamics associated with irC disease and its recovery. We longitudinally sampled stool from 38 cancer patients starting either anti-CTLA-4 or dual ICI therapy, with 13 subsequently developing irC. To investigate the causative link between the microbiome and irC, we transplanted stools of irC patients (collected before irC onset) and unaffected ICI-treated patients into colitis-susceptible ex-germ-free mice and observed exacerbated colitis in mice harboring microbiomes from patients who developed irC compared with non-irC controls. To dissect irC-microbiome associations, we performed absolute (Contijoch et al., 2019; Faith et al., 2011; Llewellyn et al., 2018; Vandeputte et al., 2021; Maghini et al., 2023) and relative abundance quantification of gut microbiome composition with both 16S ribosomal RNA (rRNA) and metagenomic sequencing. Fecal microbiomes exhibited pronounced perturbations in alpha diversity, microbiota density, and beta diversity for the first 10 days of active irC. Taxonomically, these shifts were driven by a relative expansion of Proteobacteria coupled with the contraction of other phyla, including Firmicutes. On average, these microbiota perturbations began to resolve after day 10 of active irC and were restored to a state equivalent to baseline following irC resolution. Strikingly, the acute irC microbiome phase exhibited notable parallels with previously reported characterizations of the IBD microbiota. Notably, the gut microbiome alterations mirroring IBD characteristics arise exclusively during the early inflammatory period of irC, suggesting these shifts serve as potential amplifiers and consequences of IBD pathogenesis rather than its initial trigger.

## Results and discussion

### Microbiome sampling in irC from before ICI treatment to irC resolution

**Figure 1.**
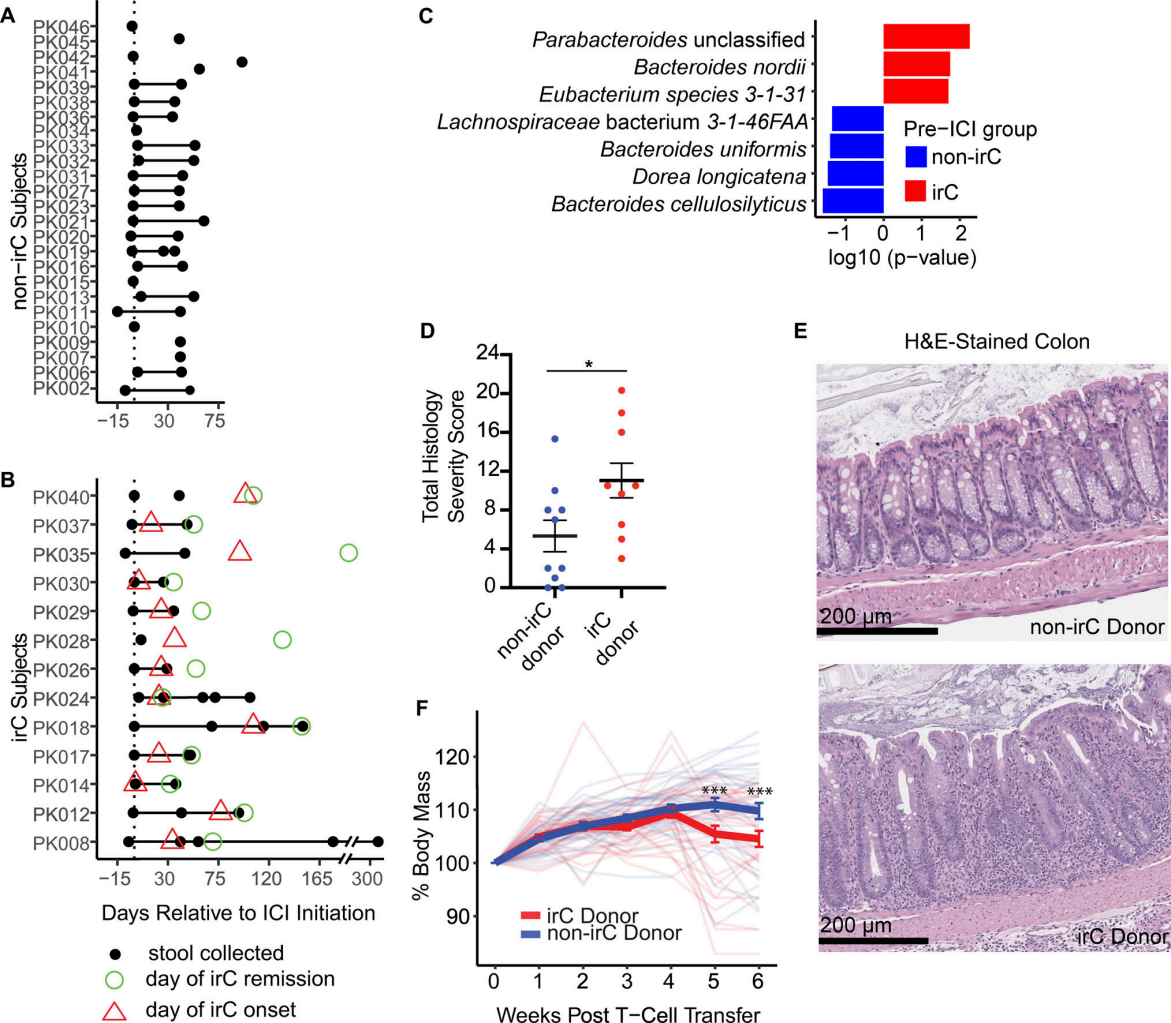
**irC patients exhibit colitogenicity in microbiome prior to irC development in a TCT colitis model. (A and B)** Stool collection (black circle) for 25 non-irC (A) and 13 irC patients (B). Day of irC onset (red triangle) and day of irC remission (green circle) are shown as days relative to the first ICI dose (vertical dotted line). **(C)** MaAsLin2 analysis (species level) examined metagenomic pre-ICI microbiome profiles of irC (red, *n* = 9) and non-irC patients (blue, *n* = 15). Features displayed are P < 0.05. The horizontal bar length indicates log_10_(P value) associated with each species. **(D and E)** Total colonic histology severity score (D) and representative H&E-stained colon sections (E) 7–8 wk after TCT in *Rag1*^−/−^ mice colonized with microbiotas from patients who would remain irC-free (blue) or eventually developed irC (red). Each dot in D represents the averaged colonic histology severity score by each microbiota donor (9 irC, 10 non-irC) (Mann-Whitney P = 0.033). **(E)** Upper: Non-inflamed colon section from the murine recipient of non-irC patient’s microbiota. Lower: Inflamed colon section from murine recipient of pre-colitis microbiota from irC patient. Moderate mucosal and submucosal inflammation with crypt and goblet cell loss, crypt hyperplasia, and muscle thickening. The horizontal bars in E represent the scale bar 200 µm. **(F)** Weekly body mass percentage change (relative to week 0) following TCT in *Rag1*^−/−^ mice colonized with microbiota from 9 irC patients (red) or from 10 non-irC patients (blue) with three to seven mice receiving a single fecal microbiota. Bolded blue and red lines represent the mean ± SEM of all irC or non-irC-colonized group of mice, respectively, at each week after TCT. Individual opaque thin lines represent the body mass change of individual mice. The linear mixed-effect model showed no significant difference in weight between the irC- and non-irC-colonized mice (P = 0.63) at week 0 but a significant effect of patient colitis status on raw weight change at weeks five and six after TCT (week 5, P = 1.7 × 10^−4^; week 6, P = 3.2 × 10^−4^). Plots D–F are combined from three independent experiments. Analysis included a total of 67 mice (experiment 1: *n* = 19, 2: *n* = 24, 3: *n* = 24) colonized with 19 human microbiotas (*n* = 10 irC patients, *n* = 9 non-irC patients). Three to seven mice colonized per microbiota donor. Error bars represent mean ± SEM. *P < 0.05 and ***P < 0.001. Linear mixed model fit by restricted maximum likelihood with P value estimations using Satterthwaite’s method “lmerModLmerTest” was performed to evaluate differences in body mass change between irC and non-irC microbiota-colonized mice groups. Mann-Whitney was performed to assess differences in colonic histological severity scores between irC and non-irC microbiota-colonized mice groups.

Given the immense resources to conduct prospective microbiome studies, virtually all microbiome association studies across all diseases rely on either cross-sectional cohorts or longitudinal post-disease cohorts. As a result, it is difficult to discern whether gut microbiota alterations are the cause or effect of disease. A prospective ICI-patient cohort enables the characterization of temporal microbiome dynamics aligned with irC clinical events, from pre-ICI initiation to post-irC remission. We enrolled 38 patients with stage III (21% 8/38 patients) or IV (79% 30/38) cancer (63% [24/38] melanoma, 13% [5/38] genitourinary, 13% [5/38] lung, 5% [2/38] mesothelioma, 2% [1/38] ovarian, and 2% [1/38] kidney) receiving anti-CTLA4–containing treatment (95%: anti-CTLA4+anti-PD1; 5%: anti-CTLA4; Tables S1 and S2). Stool samples were collected prior to ICI initiation (pre-ICI) and at follow-up (5–7 wk after ICI initiation) from all patients (Fig. 1, A and B) as part of the longitudinal cohort (LC). 13 patients (34%) developed irC, diagnosed by symptoms of diarrhea and/or colitis by Common Terminology Criteria for Adverse Events v5.0, along with endoscopic/histologic confirmation when possible (10/13, 77%). From these irC patients, we collected additional stool samples during irC and after irC remission (Fig. 1 B). The remaining 25 patients who did not develop irC served as controls (non-irC; Fig. 1 A). In total, 77 stool samples from 38 LC patients were profiled using both shotgun metagenomics and 16S rRNA gene.

### Transfer of pre-colitis onset fecal microbiome from irC patients to ex-germ-free mice can exacerbate colitis

**Figure S1.**
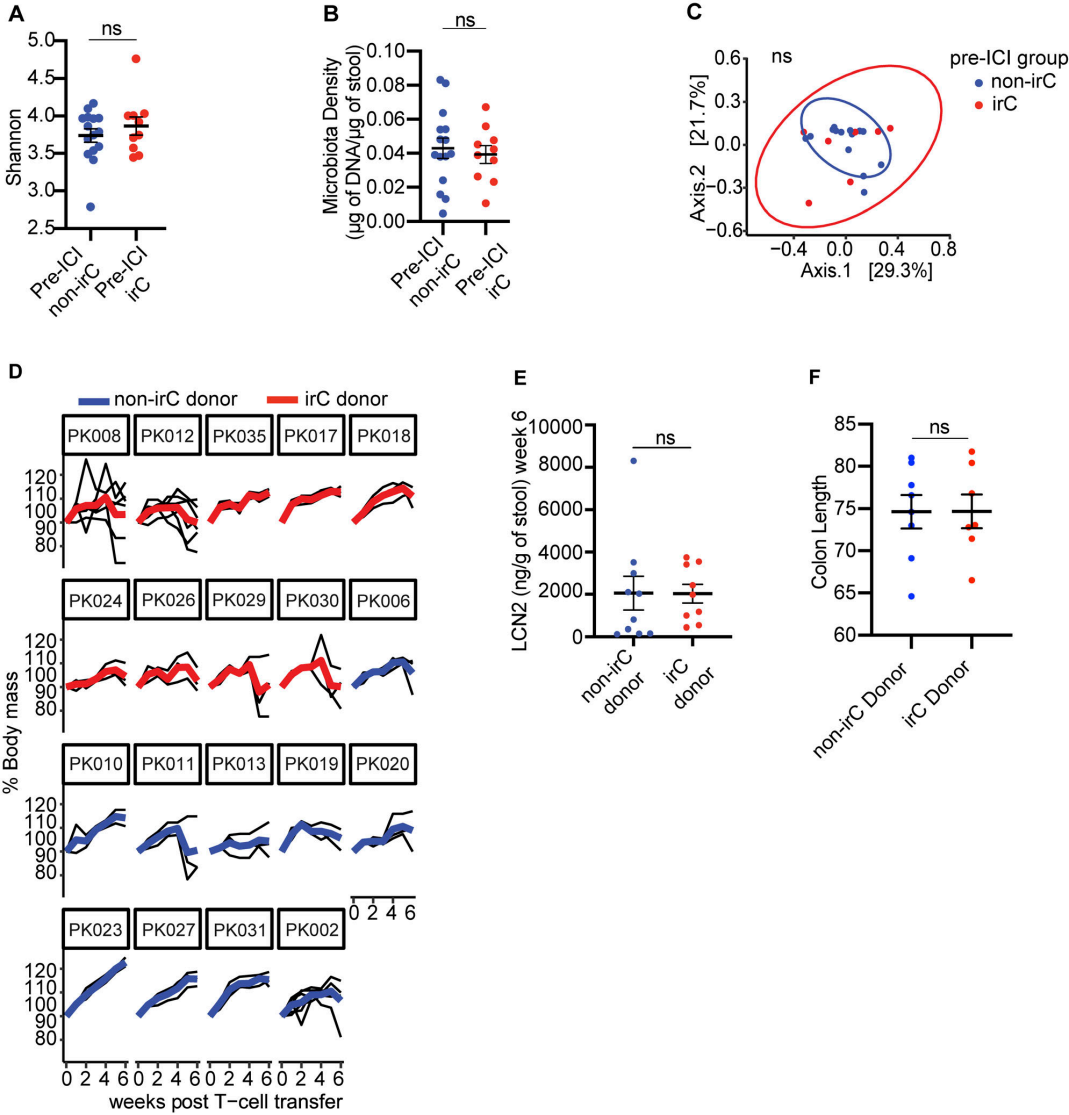
**Colitogencity in microbiomes of irC patients prior to irC development and FMT to gnotobiotic mice exacerbates murine colitis in the TCT model. (A and B)** Comparison of 16S rRNA Shannon diversity (A) and microbiota density (B) between gut microbiomes sampled prior to ICI initiation between irC (*n* = 10) and non-irC patients (*n* = 15) by Mann-Whitney (A, P = NS, B, P = NS). **(C)** PCoA plot of Bray-Curtis dissimilarity of fecal microbiota sampled prior to ICI initiation and assessed by metagenomic sequencing in patients who eventually develop irC (*n* = 10, red) or remain irC-free (*n* = 15, blue) (PERMANOVA P = 0.71). **(D)** Weekly body mass percentage (relative to week 0 weight) after TCT in *Rag1*^−/−^ mice, grouped by microbiota donor (red = irC patient, blue = non-irC patient). Presented as averaged weekly relative body mass (bolded red or blue line) of each microbiota donor and individual murine body mass (thin black line). **(E and F)** (E) Comparison of LCN2 (right, P = NS) of irC-colonized (red) versus non-irC-colonized (blue) mice 6 wk after TCT or (F) colon length (mm) at sacrifice endpoint. Individual dots represent the average by microbiota donor. Plots D–F used three to seven mice colonized per human microbiota donor, and plots D–F involved analysis of 67 mice (experiment 1: *n* = 19, 2: *n* = 24, 3: *n* = 24) colonized with 19 human microbiotas (*n* = 10 irC patients, *n* = 9 non-irC patients). Plot F used 15 microbiota donors, combined using two independent TCT experiments two and three. Error bars represent mean ± SEM. NS P >0.05 by Mann-Whitney.

To evaluate microbiome signatures associated with irC risk prior to ICI initiation, we compared 16S rRNA-based alpha diversity between non-irC and irC patients in pre-ICI microbiotas. No significant alpha diversity differences were found between irC and non-irC samples (Fig. S1 A), consistent with previous studies (Andrews et al., 2021; McCulloch et al., 2022). To assess if absolute quantities of the gut microbiota can influence irC risk, we evaluated DNA-based microbiota density, defined as μg of microbial DNA per mg of stool (Contijoch et al., 2019; Faith et al., 2011; Llewellyn et al., 2018). Similarly, no significant differences were found in pre-ICI microbiota density between irC and non-irC patient groups (Fig. S1 B). Beta diversity contrasting irC and non-irC microbiome compositions prior to ICI was not significantly different (Bray-Curtis distances, PERMANOVA P = 0.71, Fig. S1 C). To identify taxa associated with irC susceptibility, we next employed MaAsLin2 analysis on metagenomic profiles of pre-ICI irC and non-irC samples. *Eubacterium sp.*, unclassified *Parabacteroides sp.*, and *Bacteroides nordii* (all P < 0.05) were significantly enriched in the pre-ICI microbiomes of patients who eventually developed irC. *Dorea* genus, *Dorea longicatena*, *Lachnospiraceae *bacterium *3-1-46FAA*, and *Bacteroides cellulosilyticus* (all P < 0.05) were enriched at pre-ICI in non-irC patients, although non-significant after adjustment for multiple comparisons using Benjamini-Hochberg (BH) (Fig. 1 C). Previous studies on independent cohorts of anti-CTLA-4 mono- or combination-treated melanoma patients have also reported baseline enrichment of *Dorea* phylum members in patients who did not develop irC or ≥grade 3 irAEs and *Bacteroides* association with either irC susceptibility or protection depending on the species (Andrews et al., 2021; Chaput et al., 2017; Dubin et al., 2016; Usyk et al., 2021).

To investigate the potential causal link between the microbiome and irC, we employed a T-cell transfer (TCT) model of colitis (Heul and Stappenbeck, 2018; Powrie et al., 1993; Britton et al., 2019). This model simulates the loss of Treg-mediated tolerance—a key immunological feature observed with ICI—and requires gut microbes for inflammation. Unlike murine models relying on murine host microbiota or antibiotic depletion, the gnotobiotic TCT model allows direct assessment of the human microbiota’s role in colitis (Wang et al., 2018a, 2019; Heul and Stappenbeck, 2018; Zhou et al., 2023; Gao et al., 2023; Lo et al., 2023). We have previously shown that stool transfers from IBD patients into this model lead to significantly increased inflammation compared with stool transfers from non-IBD patients (Britton et al., 2019, 2020). To quantify the colitogenicity of irC microbiomes, we colonized germ-free *Rag1*^*−/−*^ mice with clarified stools from 9 irC patients sampled before irC development and 10 non-irC patients sampled at baseline. At 3–4 wk after colonization, naïve CD45RB^hi^ T cells were transferred to *Rag1*^*−/−*^ mice to induce colitis. Following 7–8 wk after colitis induction, colons were evaluated based on histological criteria (Koelink et al., 2018). Histological examination revealed that mice colonized with pre-colitis stools from irC patients demonstrated a notably enhanced severity of intestinal inflammation compared with those colonized with stools from non-irC patients (Mann-Whitney P = 0.033, Fig. 1 D, representative images in Fig. 1 E; and Tables S3 and S4). Mice colonized with stool collected from irC patients prior to irC development exhibited greater weight loss at weeks 5 and 6 compared to those colonized with stool from non-irC patients (P = 1.7 × 10^−4^ and P = 3.2 × 10^−4^, respectively; Fig. 1 F), despite similar baseline weights (P = 0.63). The difference in colitis exacerbation by non-irC and irC microbiomes is more subtle than previously observed between IBD and non-IBD gut microbiotas (Britton et al., 2019), and lipocalin and colon length differences were not significant (Fig. S1, D–F) (Chassaing et al., 2012). Taken together, this ex-germ-free microbiota transfer experiment suggests that the baseline irC gut microbiota harbors increased colitogenic potential, which may predispose individuals to irC when coupled with the immunological trigger provided by ICI therapy.

### Distinct microbiota compositions in mice reflect colitis susceptibility linked to irC patient microbiotas

**Figure 2.**
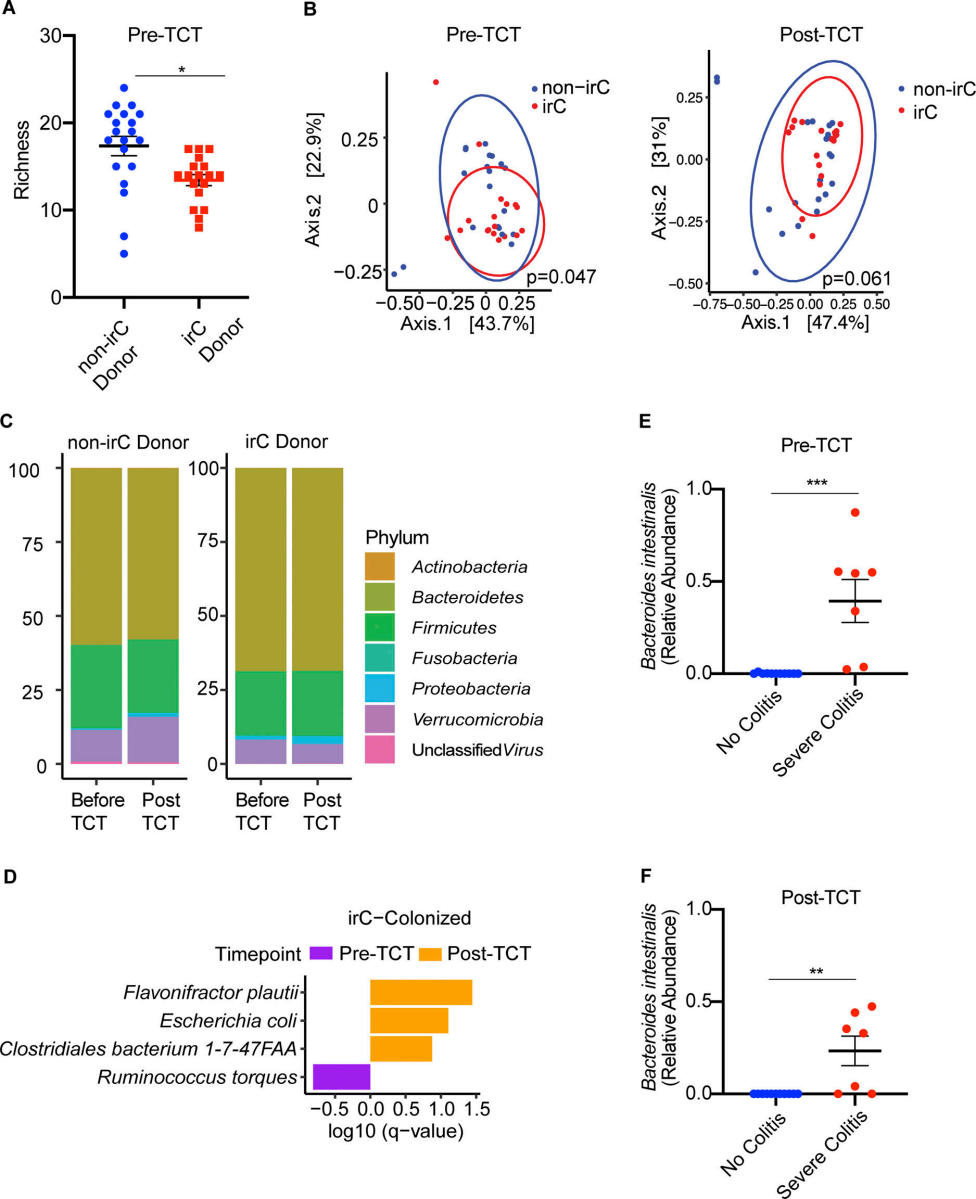
**Metagenomics of murine stool microbiome colonized with either irC or non-irC associated microbiotas.** Murine stool microbiomes were longitudinally sampled before and after TCT. **(A)** Comparison of microbiome richness before colitis induction by TCT, between mice colonized by irC (red) or non-irC (blue) microbiota (the former donor group, patient stool collected before irC development). Individual dots represent individual mice. A linear mixed model via lme4 was applied (P = 0.013) to account for the non-independence of murine samples colonized by the same patient donor. **(B)** PCoA based on Bray-Curtis distances of species-level microbial communities from murine stool samples before TCT (left) and after TCT (right). Samples are colored based on their designation as receiving microbiome from either non-irC donor (blue) or irC donor (red). The multivariate homogeneity of group dispersions was tested using PERMANOVA (P = 0.047 for pre-TCT and P = 0.061 for post-TCT). **(C)** Taxonomic composition plot of operational taxonomic units at the phylum-level pre- and post-TCT in non-irC and irC-colonized mice. Each vertical bar represents the average fecal microbiota composition of non-irC or irC donor group at the indicated timepoint (before or after TCT). **(D)** Top differentially abundant bacterial species in murine microbiomes of irC-colonized mice, either before colitis induction (pre-TCT, purple) or after (post-TCT, orange), identified by MaAsLin2. Horizontal bar length indicates log_10_(q-value) associated with each species. MaAsLin2 parameters included centered log ratio normalization and linear model analysis. Analysis included *n* = 18 mice with paired pre- and post-TCT samples. Random effects accounted for microbiota donor (to control for non-independence among mice colonized with the same microbiota donor) and mouse ID (to control for non-independence of paired samples from the same mouse). **(E and F)** Top differentially abundant bacterial species in murine microbiomes agonistic to donor source and based solely on murine colon histology severity score quartile show relative abundance of *Bacteroides intestinalis* between no colitis (blue) versus severe colitis (red) mice (E) at pre-TCT (MaAsLin2 P = 0.00048, q = 0.025) and (F) at post-TCT (MaAsLin2 P = 0.0029, q = 0.13). First quartile (orange; no colitis, score = 0); fourth quartile (green; severe colitis, score ≥14); *n* = 18 mice. Bar length indicates log_10_(q-value) associated with each taxon. MaAsLin2 parameters included centered log ratio normalization and linear model analysis. Random effects accounted for microbiota donor (to control for non-independence among mice colonized with the same microbiota donor). The study included *n* = 18 mice with paired pre- and post-TCT samples (*n* = 7 mice with severe colitis, *n* = 11 mice with no colitis). Data in A–F plots are combined from three independent TCT experiments, which used 19 human microbiotas (experiment 1: *n* = 3 human microbiotas, experiment 2: *n* = 8 human microbiotas, and experiment 3: *n* = 8 human microbiotas). From each microbiota donor, fecal samples from two mice were represented at each timepoint (pre- and post-TCT) where pre-TCT represents week 0 and post-TCT represents weeks 7–8 post-TCT to be metagenomically sequenced and analyzed. Data in plots A–D used *n* = 38 mice with paired pre- and post-TCT samples. Taxa presented in D–F had q < 0.25. Error bars represent mean ± SEM. From each microbiota donor, fecal samples from two mice were represented at each timepoint (pre- and post-TCT) where pre-TCT represents week 0 and post-TCT represents weeks 7–8 post-TCT. *P < 0.05, **P < 0.01, and ***P < 0.001. Linear mixed model fit by restricted maximum likelihood with P value estimations using Satterthwaite’s method “lmerModLmerTest.”

To evaluate microbiota composition in the gnotobiotic TCT model of irC, we metagenomically sequenced mouse fecal samples collected before and after colitis induction (Table S5). Prior to colitis induction, richness but not Shannon diversity was significantly decreased in irC-colonized mice compared with non-irC-colonized mice (Fig. 2 A, week 0, richness P = 0.013; Shannon P = 0.19). Species-level taxonomic composition significantly differed between irC and non-irC-colonized mice prior to colitis induction (PERMANOVA P = 0.047, Fig. 2 B) but not after induction (PERMANOVA P = 0.061, Fig. 2 B). We visualized phylum-level taxonomic composition (Fig. 2 C) and employed MaAsLin2 to identify bacterial species associated with pre- and post-colitis induction. In irC-colonized mice following colitis induction, *Escherichia coli* (P = 0.0038, q = 0.079), *Flavonifractor plautii* (P = 0.00088, q = 0.036), and *Clostridium *bacterium* 1-7-47FAA* (P = 0.0097, q = 0.13) were associated with post-TCT microbiomes, while *Ruminococcus torques* (P = 0.015, q = 0.15) was associated with pre-TCT microbiomes (Fig. 2 D and Table S6).

To better understand the clinical relevance of our TCT-colitis model, we next analyzed microbiome composition and colitis severity in the mice without considering the microbiota donor source. Mice were categorized based on their total colon damage histology scores, where the lowest quartile (score 0) and highest quartile (score ≥ 14) were designated as “no colitis” and “severe colitis,” respectively. MaAsLin2 analyses revealed that *Bacteroides intestinalis* was significantly enriched in the pre-TCT microbiomes of mice that would eventually develop severe colitis (week 0, MaAsLin2 P = 0.00048; q = 0.025 Fig. 2 E). At sacrifice, *Bacteroides intestinalis* remained significantly associated with severe colitis (post-TCT 7–8 wk, MaAsLin2 P = 0.0029, q = 0.13 Fig. 2 F). This is consistent with previous reports of *Bacteroides intestinalis* enrichment at baseline in combined ICB-treated patients with metastatic melanoma who eventually developed ≥grade 3 irAEs (Davar et al., 2021).

### Temporal microbiota dynamics reflect distinct clinical stages of irC

**Figure S2.**
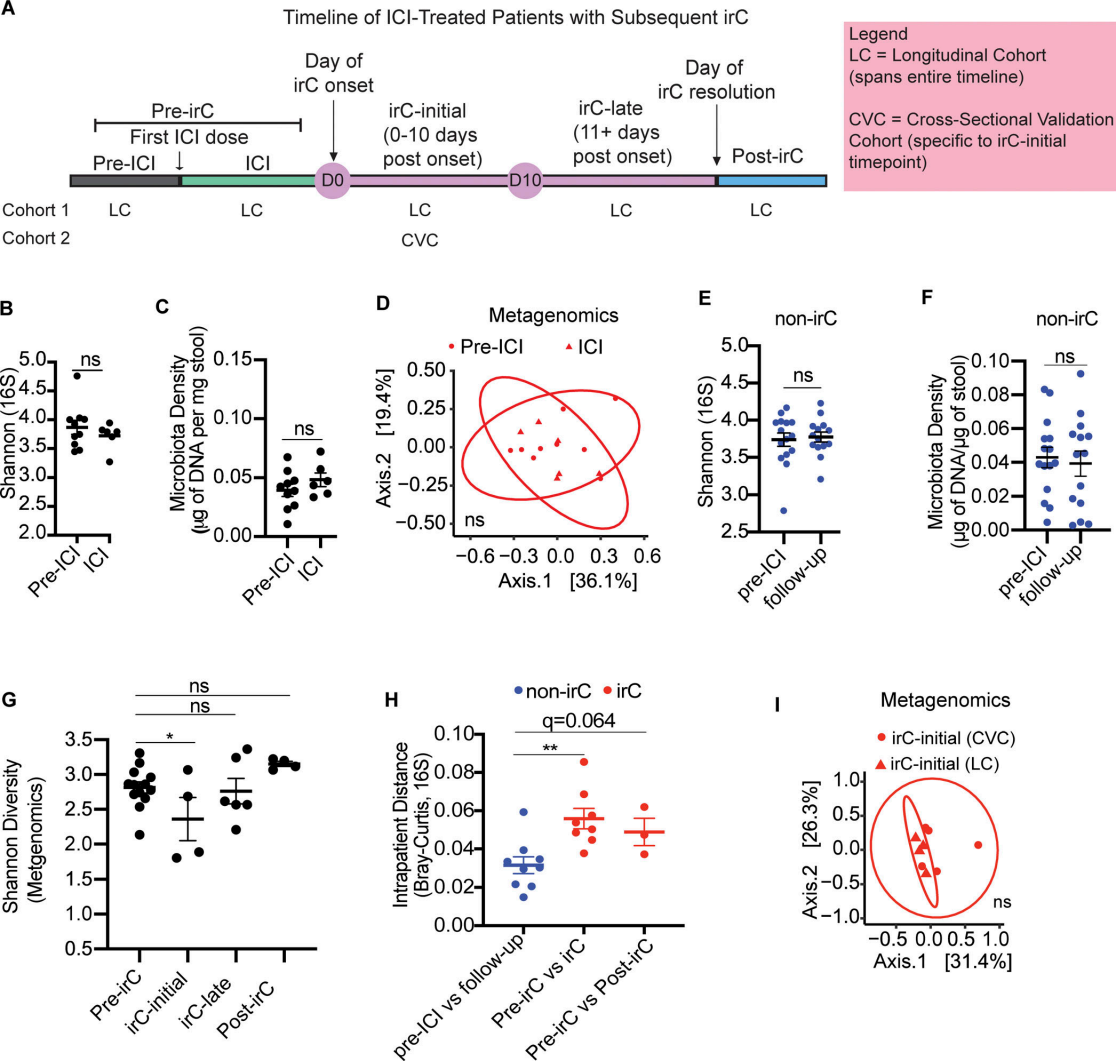
**Microbiome markers of non-irC patients and irC-patients and comparison of microbial composition with validation cohort. (A)** Schematic representation of the temporal progression of ICI-treated patients who develop irC, marked by significant clinical events: first ICI dose, irC onset, irC resolution. Timepoints are categorized into distinct segments: pre-ICI, ICI, irC-initial (0–10 days post-onset), irC-late (11+ days post-onset), and post-irC (resolution of irC symptoms). Of note, pre-ICI and ICI pooled together are referred to as “pre-irC” to represent the time segment preceding irC onset. The continuous observation of the prospective LC spans the entire timeline, from pre-ICI to post-irC. CVC is a cross-sectional validation cohort and focuses specifically on the irC-initial segment. D0 and D10 indicate the start and end of the irC-initial phase, respectively. **(B–D)** Shannon diversity using 16S rRNA sequencing data (P = NS) (B) or microbiota density (P = NS) using linear mixed model (C) or PCoA on Bray-Curtis distances of metagenomic profiles (D) to compare microbiota composition of Pre-ICI (circle) and Pre-irC microbiomes (triangle) from LC using PERMANOVA (P = NS) of irC patients microbiomes collected before ICI initiation and at ICI. **(E and F)** Shannon diversity using 16S rRNA sequencing data (E) and microbiota density (F) of non-irC microbiomes (blue) collected before ICI initiation and 5–7 wk after ICI initiation (follow-up) (E, P = NS; F, P = NS). Linear mixed model fit by restricted maximum likelihood. **(G)** Temporal Shannon diversity using metagenomic profiles at distinct irC disease stages using linear mixed model to compare Shannon values at pre-irC versus irC-initial (P = 0.015, irC-late (P = NS), post-irC (P = NS). **(H)** Microbiota profiles change (Bray-Curtis distances) over time within individuals (red = irC patients, blue = non-irC patients) using 16S rRNA sequencing data. Intrapatient comparison of microbiome in nine non-irC patients with paired stool samples collected at pre-ICI and follow-up (5–7 wk post-ICI initiation). For irC, intrapatient comparison of paired stool samples was collected at (1) pre-irC and active irC (irC-initial/irC-late) (in eight patients) (P = 0.0037, q = 0.0074) or (2) pre-irC and post-irC (in three patients, P = 0.064, q = 0.064). Mann-Whitney with BH for adjustment. **(I)** PCoA plot on Bray-Curtis distances to compare metagenomic profiles of microbiota composition by PERMANOVA (P = NS) of irC-initial microbiomes between patients from CVC and LC (red). Mann-Whitney *P < 0.05, **P < 0.01, NS P > 0.05. Linear mixed model fit by restricted maximum likelihood with P value estimations using Satterthwaite’s method “lmerModLmerTest.”

To determine the gut microbiome dynamics in irC patients, we analyzed longitudinally collected stool samples from irC patients using defined timepoint bins based on days relative to irC onset (Fig. S2 A). These bins were pre-ICI (prior to checkpoint blockade administration), ICI (post-ICI initiation but before colitis onset), irC-initial (active irC during initial 0–10 days of onset), irC-late (active irC 11+ days of onset), and post-irC (after resolution of irC). We pooled together microbiomes collected during pre-ICI or ICI as “pre-irC” (Fig. 2 A) since a comparison of samples from pre-ICI versus ICI showed that checkpoint blockade treatment did not significantly impact alpha diversity (Fig. 2 B), microbiota density (Fig. 2 C), or microbiome composition (Fig. 2 D; PERMANOVA P = 0.95).

**Figure 3.**
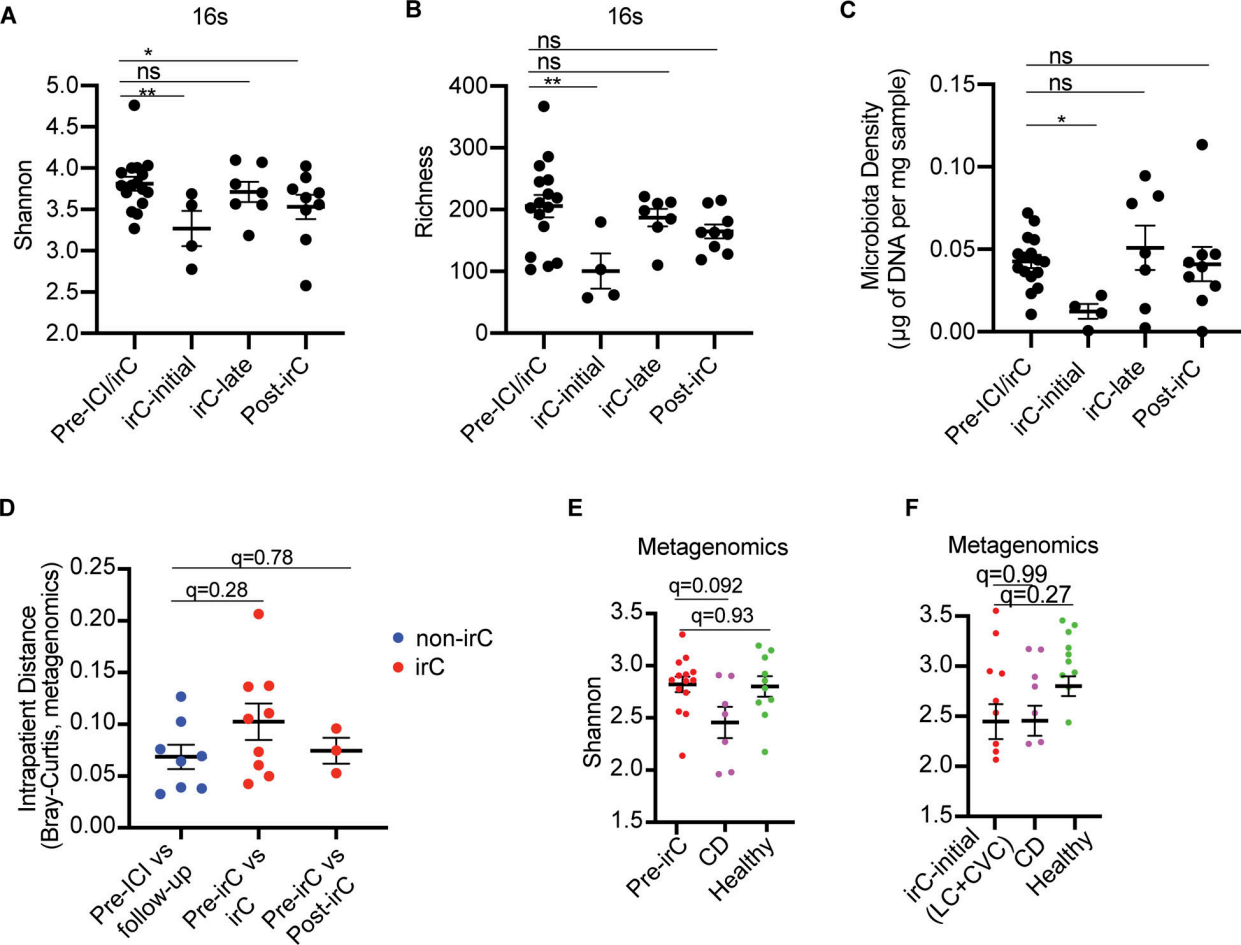
**Longitudinal sampling of patient gut microbiomes at indicated timepoints in LC cohort, along with samples from patients with CD, healthy participants, or irC patients from CVC cohort (validation). (A–C)** Longitudinal alpha diversity (A) and observed richness (B) using 16S rRNA sequencing data and microbiota density (C) at distinct irC disease stages (pre-irC, irC-initial, irC-late, and post-irC) (*n* = 13 irC patients). Linear mixed model fit by restricted maximum likelihood with P value estimations using Satterthwaite’s method “lmerModLmerTest.” Comparison of values at pre-irC versus irC-initial (A, P = 0.0042; B P = 0.0019; C, P = 0.036), irC-late (A, P = 0.039; B, P = NS; C, P = NS), post-irC (A, P = NS; B, P = NS; C, P = NS). **(D)** Intrapatient metagenomic microbiota profiles change (Bray-Curtis distance) over time within either irC patients (red) or non-irC patients (blue). Intrapatient comparison of microbiotas in eight non-irC patients (blue) with paired stool samples collected at pre-ICI and follow-up (4–7 wk post-ICI initiation). For irC microbiomes (red), intrapatient comparison of paired stool samples collected at (1) pre-irC and active irC (in nine irC patients) or (2) pre-irC and post-irC (in three irC patients). Mann-Whitney compared the dissimilarity of non-irC paired samples against that of irC paired samples (pre-irC versus irC, P = 0.14, q = 0.28; pre-irC versus post-irC, P = 0.78, q = 0.78). **(E)** Comparison of alpha diversity between pre-irC metagenomic microbiomes profiles of irC patients (red), patients with CD (purple), and healthy individuals (green). Mann-Whitney (P value) and BH adjustment (q-value) to compare alpha diversity between groups (pre-irC versus CD: P = 0.046, q = 0.092; pre-irC versus healthy P = 0.93, q = 0.93). **(F)** Comparison of alpha diversity between irC-initial metagenomic microbiome profiles of irC patients (from LC and CVC) (red), patients with CD (purple), and healthy individuals (green). Mann-Whitney (P value) and BH adjustment (q-value) to compare alpha diversity between groups (irC-initial versus CD: P = 0.99, q = 0.99; irC-initial versus healthy P = 0.13, q = 0.27). Mann-Whitney P values in E and F were adjusted for multiple comparisons (q-value) by the BH method. *P < 0.05, **P < 0.01; P value estimations using Satterthwaite’s method “lmerModLmerTest.”

Next, we compared microbiota parameters at different timepoints within irC patients (reported as P value) by applying a mixed linear model to account for multiple sampling from the same patient. Compared with pre-irC microbiomes, irC-initial microbiomes exhibited significantly decreased alpha diversity (16S rRNA sequencing, pre-irC versus irC-initial P = 0.0042; Fig. 3 A), richness (pre-irC versus irC-initial P = 0.0019; Fig. 3 B), and microbiota density (pre-irC versus irC-initial P = 0.036; Fig. 3 C) followed by a rapid and durable recovery in irC-late samples. This contrasted with non-irC patients’ stable alpha diversity and microbiota density between pre-ICI and follow-up visits in 5–7 wk after ICI initiation (Fig. S2, E and F). In metagenomic-based alpha diversity, we similarly found significantly decreased Shannon diversity and richness at irC-initial compared with pre-irC (Shannon P = 0.015; Richness P = 0.0032) with recovery to baseline levels by irC-late (Fig. S2 G). We observed a significantly higher dissimilarity in the overall microbiota composition of irC patients’ paired samples at pre-irC versus active irC compared with non-irC patients’ paired samples at pre-ICI versus follow-up in 16S profiles (P = 0.0037, q = 0.0074; Fig. S2 H) but not metagenomics profiles (P = 0.14, q = 0.28; Fig. 3 D) with the difference reaching significance in the 16S. Mice colonized with irC-initial microbiotas did not exhibit exacerbated colitis compared with those colonized with non-irC follow-up samples (total histology severity score averaged prior to statistical tests; P = 0.34). Overall, we observed that the initial acute inflammatory phase of irC is characterized by decreased microbiota density, alpha diversity, and richness coupled with increased dissimilarity from their pre-irC microbiota compositions. Importantly, these microbiota perturbations are transient, with distinct microbiota structures that reflect different clinical stages of irC. This suggests that the microbiome state during irC is reversible, contrasting with the persistent dysbiosis observed in chronic inflammatory conditions like IBD.

To validate these observations, we compared patients’ irC-initial metagenomic profiles from the LC with those from an independent cohort of ICI-treated patients with cancer and confirmed irC diagnosis (five patients, cross-sectional validation cohort [CVC]; Fig. S2 A) (Elkrief et al., 2023). The irC-initial microbiota composition between LC and CVC exhibited considerable overlap by Bray-Curtis distances (PERMANOVA P = 0.38, Fig. S2 I).

To explore parallels between the irC and IBD, we compared microbiomes of irC patients to those of Crohn’s Disease (CD) and healthy patients from previously published cohorts (Table S7) (Canales-Herrerias et al., 2023; Aggarwala et al., 2021). We found that pre-irC microbiomes had similar Shannon diversity to healthy individuals (P = 0.93, q = 0.93; Fig. 3 E) but significantly different from CD microbiomes (P = 0.046, q = 0.092; Fig. 3 E). In contrast, irC-initial microbiomes showed Shannon diversity comparable with CD microbiomes (Mann-Whitney P = 0.99, q = 0.99; Fig. 3 F). These results suggest the gut microbiome during irC-initial represents an IBD-like state that transiently occurs after ICI therapy, with the resolution even prior to the completion of the irC clinical course. This transient IBD-like microbiome state may have implications for understanding the mechanisms underlying irC and for developing microbiome-targeted interventions.

### Expansion of pro-inflammatory bacteria and loss of beneficial bacteria during irC-initial

**Figure 4.**
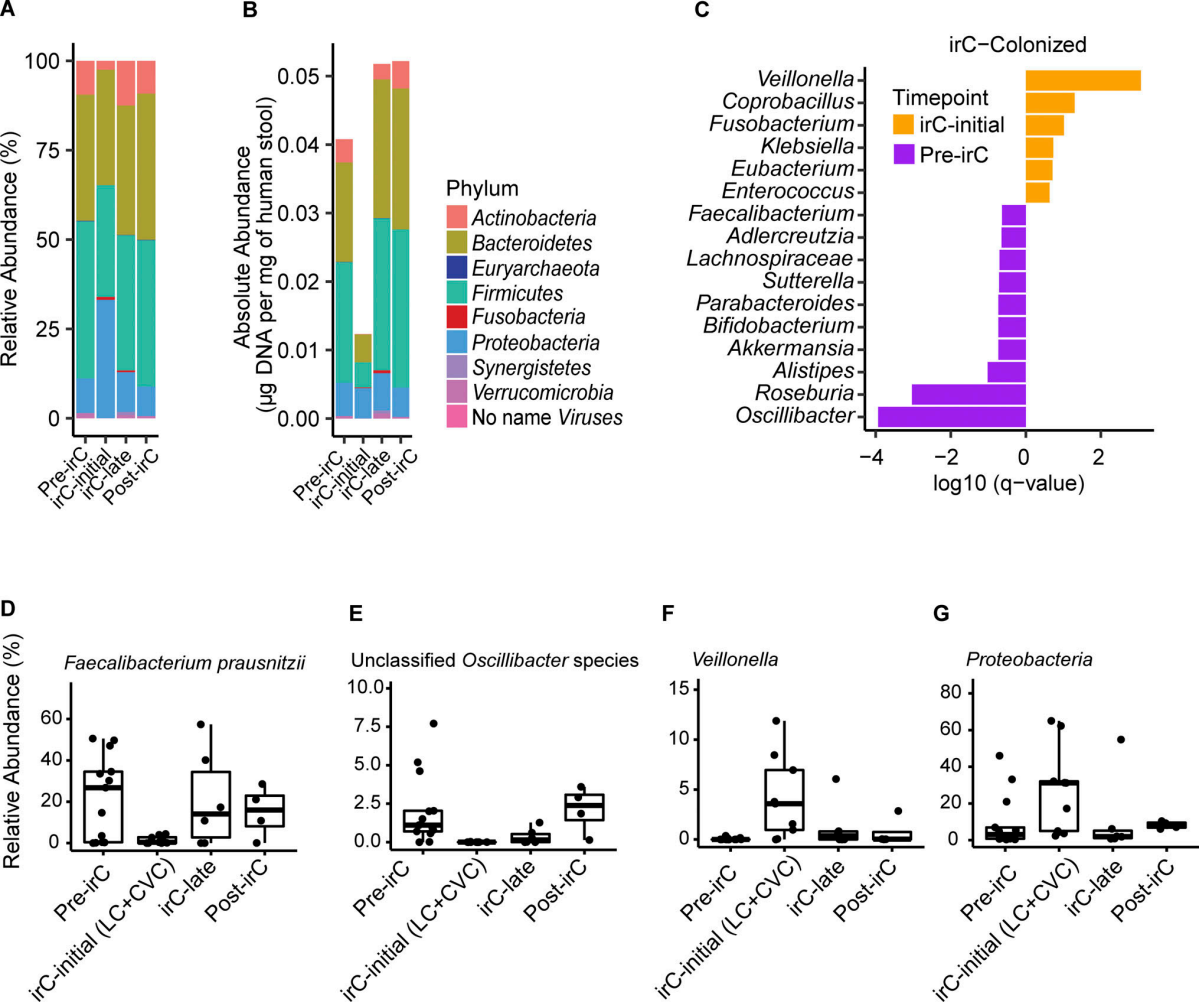
**Fecal microbial taxonomic signatures are differentially associated with distinct stages of irC. (A and B)** Longitudinal taxonomic profiling of irC patients’ microbiomes at pre-irC, irC-initial, irC-late, and post-irC in terms of (A) relative abundance of operational taxonomic units at the phylum-level by metagenomic profiling and (B) absolute abundance. **(C)** Top bacterial genus associated with pre-irC versus irC-initial identified by MaAsLin2 using metagenomically sequenced microbiomes of irC patients at pre-irC (orange, *n* = 13) and irC-initial (purple, *n* = 9 where *n* = 4 from LC and *n* = 5 from CVC). Random effect adjusted for non-independence of repeated measures. The horizontal bar length indicates log_10_(q-value), in which q-value was calculated by BH adjustment of P value. Genus presented q < 0.25. MaAsLin2 parameters included centered log ratio normalization and linear model analysis. **(D–G)** Temporal dynamic changes in the relative abundance of (D) *Faecalibacterium prausnitzii*, (E) unclassified *Oscillibacter* species, (F) *Veillonella* genus, (G) Proteobacteria phylum in irC patients at pre-irC, irC-initial, irC-late, and post-irC. irC-initial samples were pooled from both irC cohorts (LC and CVC). Based on metagenomic profiles. Each symbol represents data from an individual patient; the boxplot displays a central line presenting the median, accompanied by a box that encloses the interquartile range (IQR) and extends whiskers up to the farthest data point within 1.5 times the IQR.

**Figure S3.**
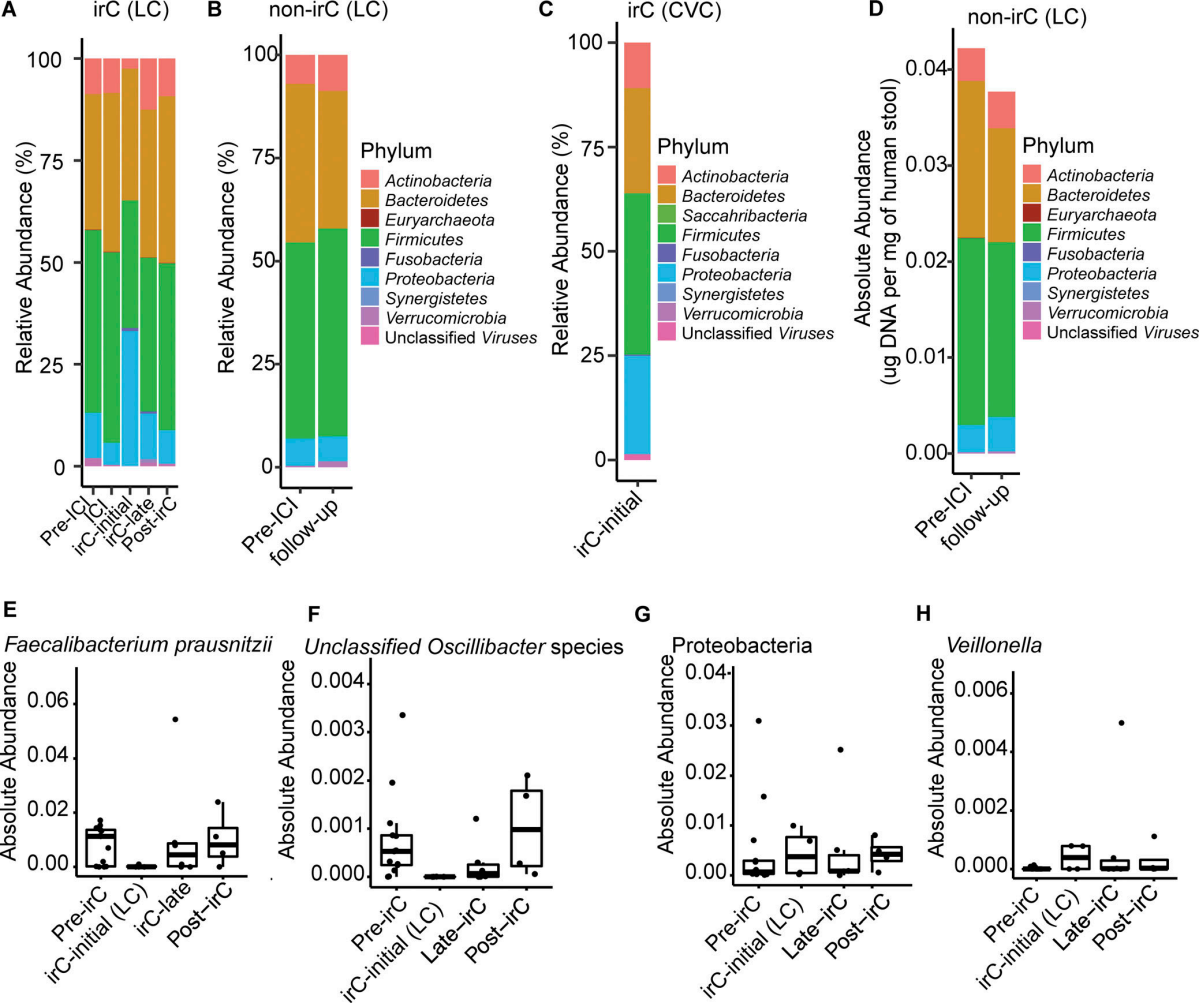
**Taxonomic composition at phylum-level and absolute abundance of select taxa in irC and non-irC patients. (A–C)** Relative abundance of operational taxonomic units at the phylum level (A) of irC patients from LC at five timepoints (pre-ICI, ICI, irC-initial, irC-late, and post-irC) or (B) of non-irC from LC at pre-ICI and follow-up (5–7 wk after the first dose of ICI) or (C) irC-initial gut microbiomes from irC patients of validation cohort, CVC. Each bar represents the average fecal microbiota composition at the indicated timepoint within a patient group. **(D)** Absolute abundance at phylum level of non-irC patients at pre-ICI and follow-up (5–7 wk after the first dose of ICI). **(E–H)** Temporal dynamic changes in the absolute abundances of (E) *Faecalibacterium prausnitzii,* (F) unclassified *Oscillibacter* species, (G) Proteobacteria phylum, and (H) *Veillonella* genus in irC patients of LC at pre-irC, irC-initial, irC-late, and post-irC relative abundance from metagenomic profiles and microbiota density were used to calculate each taxa’s absolute abundance. Each symbol represents data from an individual patient; boxplot displays a central line presenting the median, accompanied by a box that encloses the interquartile range (IQR) and extends whiskers up to the farthest data point within 1.5 times the IQR.

In irC patients, we observed striking temporal taxonomic shifts at irC onset, including a relative expansion of Proteobacteria and reduction of other phyla such as *Actinobacteria*, *Firmicutes*, and *Verrucomicrobia* (Fig. 4 A and Fig. S3 A). Conversely, non-irC phylum composition remained relatively stable over time (Fig. S3 B). Proteobacteria relative abundances in irC-initial samples were similarly elevated in both CVC and LC (LC versus CVC Proteobacteria Mann-Whitney P = 0.56, Fig. S3 C). To determine whether Proteobacteria expansion was due to increased absolute abundance or decreased absolute abundance of other phyla, we evaluated the temporal changes in each phylum’s absolute microbiota density. At irC-initial, we observed that the absolute abundance of Proteobacteria remained constant while that of all other phyla decreased (Fig. 4 B), consistent with previous observations of decreased absolute bacterial abundances in IBD microbiomes (Contijoch et al., 2019). Recovery of many phyla could be observed at irC-late (Fig. 4 B), despite unresolved colitis. By contrast, non-irC patients’ absolute microbiota density remained stable (Fig. S3 D).

To further probe microbiota shifts at irC onset, we applied MaAsLin2 analysis to compare metagenomics profiles between pre-irC microbiomes (from LC) and irC-initial microbiomes (from CVC/LC). In irC-initial microbiomes sampled from both cohorts, MaAsLin2 identified taxa candidates at the level of the genus (Fig. 4 C and Table S8) and species (Table S9) that were more abundant at pre-irC compared with irC-initial. We observed a reduction of numerous bacterial genera at irC-initial compared with pre-irC, (Fig. 4 C and Table S8), which included species (Table S9) such as unclassified *Oscillibacter* species (q = 0.00021), *Roseburia intestinalis* (q = 0.015), *Akkermansia muciniphila* (q = 0.21), and *Faecalibacterium prausnitzii* (q = 0.27). *Faecalibacterium prausnitzii* and unclassified *Oscillibacter* species were then recovered by irC-late in terms of both relative (Fig. 4, D–G) and absolute abundance (Fig. S3, E–H). *Faecalibacterium prausnitzii* is frequently reported to be an important constituent of healthy microbiota composition (Yatsunenko et al., 2012). For example, *Faecalibacterium praustnizi* is an important short-chain fatty acid producer that has been widely reported to be significantly decreased in IBD patients (Machiels et al., 2014; Sokol et al., 2009) and has also been negatively associated with ICI-treated patients who developed colitis (Gao et al., 2023). In a prospective study, stool samples were collected between 2008 and 2017 from 3,483 healthy first-degree relatives of patients with CD (Raygoza Garay et al., 2023). Garay et al. studied the pre-disease microbiome of 73 of these individuals who subsequently went on to develop CD and found *Oscillospiraeceae*, *Roseburia*, and *Faecalibacterium* among the top CD-predictive taxa, and their decreased abundances correlated with risk of CD development in at-risk healthy relatives. We also observed depletion (relative to baseline abundances) of these three taxa during irC-initial in our cohort.

Conversely, bacterial genera associated with irC onset (compared to pre-irC) (Fig. 4 C and Table S8) included *Veillonella*, *Coprobacillus*, *Fusobacterium*, *Klebsiella*, *Eubacterium*, and *Enterococcus* genus. Specific species such as *Veillonella atypicae* (q = 0.024), *Veillonella parvula* (q = 0.038), *Veillonella unclassified* (q = 0.041), *Veillonella dispar* (q = 0.15), and the Proteobacterium *Klebsiella pneumoniae* (q = 0.17) were elevated (Table S9). By late-irC, the relative and absolute abundances of *Veillonella* and *Proteobacteria* returned to initial pre-irC levels (Fig. 4, F and G; and Fig. S3, G and H). Prior to irC, the relative abundance of Proteobacteria (median, 3.1%) is comparable with that of healthy microbiota (Shin et al., 2015), but expands (median, 31.2%) during irC-initial. Expanded gut colonization by oral pathobionts such as *Veillonella dispar*, *Veillonella parvula,* and *Klebsiella pneumoniae* has been reported in IBD patients (Schirmer et al., 2018; Ananthakrishnan et al., 2017; Tiwana et al., 2001; Atarashi et al., 2017). It has been proposed that *Veillonella* and *Klebsiella* species expand during gut inflammation because of their adaption to the damaged gut’s more oxygenic atmosphere, radical oxygen species (ROS) presence, and altered inflammation-associated metabolism (Rojas-Tapias et al., 2022). *Veillonella* species can utilize a respiratory nitrate reductase to fuel its growth with nitrate, a metabolite more abundant during inflammation (Rojas-Tapias et al., 2022). This may explain why we observed *Escherichia coli* expansion in irC-colonized mice after colitis induction and can use inflammation byproducts such as nitrate and ROS (Winter et al., 2013). Members of the Protobacteria phylum and *Enterobacteriaceae* family, *Escherichia coli* and *Klebsiella*, were enriched in irC-colonized mice and irC patients after irC onset, respectively.

### Summary

This study’s prospective irC cohort and preclinical model evidence the microbiome’s causative nature in irC and demonstrate a clear trajectory of microbiome changes before, during, and after irC development. This colitogenicity of the irC microbiome prior to irC development suggests an individual’s gut microbiome could predispose them to colitis when coupled with a triggering event like anti-CTLA4 therapy. Our findings also highlight the parallels between IBD and irC microbiome composition, suggesting that irC may represent a transient IBD-like state induced by ICI therapy. Identifying these parallels could advance our understanding of their etiology and set the stage for disease prevention and treatment strategies. While both IBD and irC may respond to immunomodulatory therapies as well as microbiota manipulation via FMT (Wang et al., 2018b; Elkrief et al., 2023; Wang et al., 2020; Halsey et al., 2023; Haifer et al., 2022; Paramsothy et al., 2017; Costello et al., 2019; Rossen et al., 2015; Moayyedi et al., 2015), these observations of the differential microbiome composition across diseases may provide insights into why long-term therapy appears necessary for management of IBD but less commonly for irC. Further probing of these differences for both the microbiome and the immune system could yield important insights into the shared and discrepant features of intestinal inflammatory disease to inform therapeutic strategies.

## Materials and methods

### Study design and participants of the LC

Individuals with active cancer beginning anti-CTLA-4 or dual anti-CTLA4+anti-PD-1 treatment were prospectively enrolled at the Memorial Sloan Kettering Cancer Center (MSK) as part of this study between December 2020 to present. The research was conducted in accordance with the Institutional Review Board–approved research protocol. Clinical data were collected under protocol number 20-427. Upon identification of suspected irC, patients were referred for urgent evaluation with a study gastroenterologist. Diagnosis of irC was made using a combination of clinical assessment and exclusion of alternative etiologies, as well as endoscopic and histologic confirmation of irC when feasible. Clinical remission of irC was determined by the resolution of clinical symptoms.

Biospecimen sampling for all patients enrolled in the study included prospective stool collection at baseline before ICI infusion and at 5–7 wk follow-up after ICI initiation (follow-up). Upon the occurrence of irC, an additional collection of stools was conducted, and then the collection was again requested at irC remission. Extensive clinical metadata including demographics, cancer details, irC details, and treatment regimens were also collected for each patient.

For both irC and non-irC patients, stools were analyzed as “pre-ICI” if stool collection occurred ≤0 days relative to the first ICI dose. For follow-up samples of non-irC patients, only stools collected 5–7 wk post-ICI dose were analyzed for consistency. For categorization of irC stools relative to irC disease course, the following timepoint bins were made: (1) pre-ICI (if stool collection date occurred day ≤0 days relative to ICI initiation, (2) ICI (if stool collection occurred after ICI initiation but before irC onset), (3) irC-initial (active colitis 0–10 days), (4) irC-late (active colitis 11+ days), (5) post-irC (irC resolution). We also referred to samples collected during pre-ICI and ICI as “pre-irC” to refer to gut microbiotas collected prior to irC onset.

### LC stool sample collection and processing stool collection and DNA extraction

Fecal samples from patients were prospectively collected at either MSK clinical facilities or at home. If collection occurred at the patient’s home, samples were packed immediately with chill packs (that were frozen at least 12–24 h before sample collection) in styrofoam coolers to be sent to MSK. Patients were instructed to contact a clinical coordinator as soon as possible to arrange sample delivery. Patients were instructed to collect stool samples Monday–Thursday to ensure staff availability to coordinate sample collection. At the MSK Molecular Biology Facility, stool samples were aliquoted into cryovials and frozen at −80°C after collection at the Molecular Biology Facility. Using same-day delivery, stool specimens were kept on dry ice in an insulated box until arrival at the Mount Sinai Microbiome Translational Center (MTC) for storage and preprocessing. Upon arrival at the MTC, samples were subaliquoted on liquid nitrogen before storage at −80°C until further processing. To enable DNA quantification, each fecal sample aliquot size was targeted in the linear range of fecal DNA protocol, which is ∼20–200 mg in humans. Microbial DNA extraction from stool was conducted using previously described methods that included bead beating in phenol:chloroform (Contijoch et al., 2019; Britton et al., 2019). Quantification of purified DNA was performed using Broad Range Quant-IT dsDNA Assay Kit alongside a BioTek Synergy HTX Multi-Mode Reader. We conducted a controlled reproducibility study by processing and sequencing a subset of 28 samples (from either irC or non-irC patients), consisting of 2 aliquots each from 14 different stool samples from our patient cohort, at separate times. The aliquots from the same sample were delivered from MSK to MTC ∼9 mo apart. The stool aliquots from batch 1 and batch 2 were processed separately, ∼2 mo apart. PCoA demonstrated a close clustering of metagenomic sequences from the different aliquots of the same microbiota sample across both batches, indicating minimal experimental variation with no significant difference by batch (Adonis P value = 1.0).

### 16S rRNA sequencing

Samples were then prepared for metagenomic shotgun and 16S rRNA sequencing. For 16S rRNA sequencing, we used custom-barcoded primers that target and amplify the V4 variable 16S rRNA region to generate Illumina sequencing libraries as previously described (Faith et al., 2013). The amplified product was then purified with Beckman Coulter AMPure XP beads. Prepped libraries were sequenced on an Illumina MiSeq V2 platform as per the manufacturer’s instructions. Raw 16S rRNA amplicon sequences were processed with DADA2 software package’s built-in algorithms, which included the correction of amplicon sequencing errors (Callahan et al., 2016). SILVA 16S rRNA sequence database served as a reference for pair-end reads merge and alignment (Quast et al., 2013). The output is an amplicon sequence variant table. The sequence data files (FASTQ) for all sequencing samples are stored in the Sequence Read Archive (SRA) under project number PRJNA1012329 (Table S10).

### Metagenomic shotgun sequencing

Metagenomic libraries were prepared with a NEBNext Ultra II DNA Library Prep Kit, as previously described (Callahan et al., 2016). DNAse fragmented DNA were then subjected to end repair, Illumina adaptor ligation, and purified/size selected with Beckman Coulter SPRI beads. We then amplified the ligated product using custom i5 and i7 index primer using NEBNext Ultra Q5 Master Mix. The final products were quantified, pooled, purified with Beckman Coulter AMPure XP beads, and finally sequenced using an Illumina HiSeq 4000. For mouse microbiome samples, sequencing libraries were generated with the plexWell library preparation kit. MetaPhlAn2 was used to assign taxonomy and estimate microbial taxonomic relative abundance (Segata et al., 2011). The output is a MetaPhlAn2 relative abundance taxonomic profile. The sequence data files (FASTQ) for all sequencing samples are stored in the SRA under project number PRJNA1012329 (Table S10).

### Microbiome analysis

Previously published metagenomic microbial profiles, along with associated clinical metadata, were sourced from the SSRA and from the original authors. These sequences were derived from healthy individuals, patients diagnosed with CD, and from the CVC of irC. Specifically, for the CVC (Elkrief et al., 2023), we only included samples from irC patients that were collected within the first 10 days of active irC manifestation. These metagenomic samples were subsequently pooled and analyzed using MetaPhlAn2. The average raw read counts for human metagenomic samples from the LC, CVC, healthy cohorts, and those with CD was 3,291,931 reads/sample. Healthy and CD samples were from three metagenomic sequencing runs, so we assessed for batch effects by evaluating clustering by batch. We found minimal signs of batch effects across runs as there was a lack of clustering by batch on PCoA (Adonis P value = 0.36). For the murine metagenomic samples, the average read count was 1,187,003 reads/sample. Meanwhile, the human 16S rRNA samples from the LC patient cohort had an average of 54,939 reads/sample. If multiple intrapatient samples were collected during a single timepoint bin being analyzed (e.g., two samples collected at irC-initial from a single individual), then values were either averaged to ensure equal weighting by subject in the microbiome analysis or if using mixed-effects models, adjusted for within-patient correlations through random effects. For consistency across patients, in patients who developed recurrent episodes of irC, we focused our analysis on the first episode. Downstream analysis and visualizations from both metagenomic and 16S rRNA sequencing were created in R statistical software (RStudio Team, 2020). R packages utilized for both 16S rRNA and sequencing analysis and visualizations included *phyloSeq* (McMurdie and Holmes, 2013), *vegan* (Oksanen et al., 2023), *ggplot2*, *tidyverse*, *dyplr*, and *ggpubr*. To remove low abundance taxa that are potential contaminants, we removed ambiguously annotated phylum (e.g., “NA”) and filtered out taxa present in <2% of samples. Next, we retained taxa present above 0.1% relative abundance in at least one sample. For consistency, this filtering threshold was uniformly applied to murine samples and human samples from all cohorts. We employed a Bray-Curtis distance matrix to generate intrapatient distances between paired samples at different timepoints. Inter-patient Bray-Curtis distances were calculated on taxa relative abundance and compared using PERMANOVA with 999 permutations. Visual representation was generated on PCoA of Bray-Curtis distances of taxon-relative abundances. Additionally, taxonomic composition plots were generated based on average relative abundance of phylum between groups or timepoints within groups. MaAsLin2 was used to identify significant taxa while adjusting for potential confounders and linear model as an input parameter. Alpha diversity and observed richness (the number of operational taxonomic units observed at least once in a sample) were calculated on raw, unfiltered data. Observed richness was calculated using *estimate_richness*, and alpha diversity was calculated following the Shannon alpha diversity formula:

$$-\overset{S}{\underset{i=1}{\sum }}P_{i}\ln\left(P_{i}\right)$$

where *P*_*i*_ is the proportion of total sample represented by taxa *i* and *S* is the number of taxa (Shannon, 1948).

### DNA-based microbiota density

To understand how population density within a microbial ecosystem could influence irC, we utilized microbiota density by using previously established methodologies to process stool samples that have been shown to be unaffected by fecal water content (Contijoch et al., 2019; Faith et al., 2011; Llewellyn et al., 2018). Microbiota density was defined as the ratio of the total DNA (in μg) extracted from each stool sample to the total mass of the sample (in mg). To compute the microbial taxa’s absolute abundance, we multiplied the microbiota density measurement by the microbial taxa’s relative abundance in a sample.

### Gnotobiotic mice

Under strict sterile conditions, germ-free C57BL/6J and C57BL/6J *Rag1*^−/−^ mice were bred in flexible vinyl isolators at the Mount Sinai Precision Immunology Institute Gnotobiotic Facility. After weaning, germ-free mice were aseptically transferred outside of breeding isolators for human microbiome colonization. A single oral gavage delivered 200 μl of a fecal slurry prepared from a human stool sample. Following colonization, mice were housed in autoclaved, filter-top cages. In addition, all bottles, food, and drinking water were autoclaved before use. All animal studies presented in this study were approved by the Institutional Animal Care and Use Committee of Icahn School of Medicine and were performed in accordance with approved animal experimentation guidelines at the Icahn School of Medicine at Mount Sinai.

### Human fecal samples preparation for oral gavage

Under strict anaerobic conditions, ∼400 mg of pulverized stool was blended into a fecal slurry, using previously described methods, passed through sterile 100 μm strainers for debris removal, and diluted 1:20 in LYBHIV4 media with a final concentration of 15% glycerol (Britton et al., 2020). Fecal slurries were stored at −80°C until needed for oral gavage.

### TCT gnotobiotic colitis model

TCT experiments were conducted as previously described (Britton et al., 2019, 2020). Briefly, splenic naive CD4 T cells (CD45RB^hi^, CD25^−^) were isolated from 7–9-wk-old donor-specific pathogen-free C57BL/6J mice (The Jackson Laboratory) after tissue dissociation. Negative selection magnetic beads (Magnisort; eBioscience) were utilized to enrich for red cell blood lysis CD4^+^ T cells. Following enrichment, cells were stained for CD4, CD25, and CD45RB. Flow cytometry was utilized to sort the cell fraction of interest at a purity of at least 98%. Sorted cells were washed three times with sterile PBS before intraperitoneal injection into recipient *Rag1*^−/−^ mice. Each mouse received 1 × 10^6^ CD45RBHi T cells in 200 μl of sterile PBS. Donors and recipients were sex-matched (Table S10).

Prior to TCT (analyzed as “week 0”), receipient mouse’s body weight and fecal pellets were collected. Following colitis induction by TCT, weekly collection of body weight occurred. We collected fecal pellets starting at week 4. Any mouse that died before week 3 was excluded from the analysis. Any mouse that died at or after 3 wk prior to the experimental endpoint had their data carried forward and included in the data for subsequent weekly timepoints. The experimental endpoint was either week 7 or 8 after TCT induction at which stool and colon samples were collected. Stool samples were stored at −80°C until further processing so that shallow metagenomics could be performed on fecal pellets to determine gut microbiota composition and analyzed as described above for metagenomic sequences. Stool samples were also used to quantify lipocalin2 (LCN2) levels. LCN2, colon length, and histology scores were averaged by microbiota donor source prior to statistical analysis.

### Lipocalin

Intestinal inflammation was assessed using LCN2 concentrations in fecal samples. Sterile tubes, pre-weighed and barcoded, were used for collecting fecal pellets. These samples were then stored at −20°C until needed for analysis. For analysis, each pellet was weighed and mixed with sterile PBS in a volume (in μl) that corresponded to 10 times the pellet weight (e.g., 10 mg pellet was resuspended in 100 μl of PBS) before being homogenized with a BeadBeater for 2 min without any beads present. After this, the tubes underwent centrifugation at 4,000 rpm for a duration of 20 min. The supernatant obtained was then used for sandwich ELISA (sourced from R&D systems) to determine the LCN2 levels. These concentrations were subsequently adjusted based on the initial fecal weight. For controls, we utilized stool derived from an IL10^−/−^ mouse with known colon tumors that had been shown to have high lipocalin levels consistently across different assay operators.

### Colon collection and histology

For the TCT gnotobiotic model, the colon was dissected from the animal at the experimental endpoint. Upon tissue collection, fixation occurred in 10% buffered formalin for 24–48 h before transfer to 70% ethanol. Paraffin embedding, serial sectioning, glass placement, and Hematoxylin and Eosin (H&E) staining were conducted at HistoWiz Inc. Slides were then scanned to create whole slide image and then evaluated by a board-certified gastrointestinal pathologist in a blinded manner. Sections of colons from the TCT experiment were scored according to histological criteria (Tables S3 and S4) selected by the pathologist, based on a previous study (Koelink et al., 2018). Eight histological components were assessed: inflammatory infiltrate, goblet cell loss, hyperplasia, crypt density, muscle thickness, submucosal infiltration, ulceration, and crypt abscesses (all scored from 0 to 3). A total histological severity score, ranging from 0 to 24, was obtained by summing the eight-item scores. For FMT donor source-agonistic analysis, we calculated the quantiles of total colon histology scores using the *quantile* function in R and filtered the dataset to retain only the samples in the lowest and highest quantiles based on their total histology severity scores.

### Statistical analysis

Statistical analysis was performed using R Statistical Language (version 4.0.5) or Prism 8 (GraphPad). Wilcoxon or Mann–Whitney test was performed for cross-sectional intergroup comparisons of microbiome profiles when appropriate where P < 0.05 threshold was applied for statistical significance with two-sided comparisons unless otherwise indicated. For beta diversity analysis using Bray-Curtis distances, we calculated PERMANOVA values using *adonis* from the *vegan* (Oksanen et al., 2023) package with 999 permutations. Longitudinal and intragroup comparisons were analyzed using linear mixed effects models while adjusting for potential confounders. For example, non-independence between samples was accounted for using a mixed-effect linear model with either *lme4* or with *MaAsLin2* for microbiome taxonomic features.

For cross-sectional intergroup comparisons for TCT analysis (e.g., Shannon diversity at a single timepoint), we used the following model:

measurement ∼ human_donor_colitis_status+(1| donor_microbiota)

that accounts for the non-independence between replicate mice colonized with the same donor microbiota.

To calculate differences in weekly weights between mice colonized with either irC and non-irC-associated microbiotas, we applied a linear mixed-effect model (*lmer* model) using *lme4* and *lmerTest* in R, where linear mixed model fit by restricted maximum likelihood with P value estimations using Satterthwaite’s method *lmerModLmerTest*. The response variable was modeled using the following formula:

$$\text{measurement}\;∼\;\text{human}\_\text{donor}\_\text{colitis}\_\text{status}*\text{week}+\left(1|\;\mathrm{donor}\_\text{microbiota})+\left[1\right|\left(\text{donor}\_\text{microbiota}/{\text{mouse}}_{\text{id}}\right)\right]$$

where fixed effects specified the interaction between human donor colitis status and the week of weight collection to assess how the colitis status of the human donor and the week affect the measurement. Random effects included a random intercept for microbiota to account for variability due to different microbiota donor stools colonized in multiple mice. Additionally, a nested random intercept at the level of individual mice within each microbiota donor group accounted for the non-independence of measurements taken from the same mouse over time.

For intragroup comparisons (e.g., longitudinal Shannon diversity in irC patients only), we applied the model:

$$\text{measurement}\;∼\;\text{time}\;\text{point}+\left(1|{\text{patient}}_{\text{id}}\right)$$

to account for the non-independence of repeated measures from the same individual.

In both mouse and human analysis, MaAsLin2 was applied to identify associations between microbiome features and metadata with random effects when appropriate to de-confound non-independence among mice with the same patient microbiota or if repeated measures were taken from the same mouse or patient. MaAsLin2 adjusts for multiple hypothesis testing using the BH procedure. The statistical significance threshold for q-value was q < 0.30. Prior to MaAsLin2 application, we agglomerated to the taxa-level of interest (either genus or species level) and added a pseudo-count to each feature by calculating two orders of magnitude below the minimum non-zero value in the dataset. Center log-ratio transformation was applied to the pseudo-count-adjusted data to normalize the data by scaling each feature relative to the geometric mean of all features in the sample, facilitating the comparison across samples (Gloor et al., 2017).

### Online supplemental material

Fig. S1 shows pre-ICI microbiome comparisons (Shannon diversity, microbiota density, and beta diversity) between irC versus non-irC patients and supporting data for colitogenicity in baseline microbiome composition via FMT to ex-germ-free mice and exacerbation of murine colitis in the TCT model. Data in panels A–C are supplemental to Fig. 1, A–C, and data in panels D–F are supplemental to Fig. 1, D–F. Fig. S2 shows the schema of indicated timepoints used for analysis in ICI-treated patients with subsequent irC development and analysis of microbiome markers of non-irC patients and irC-patients, with a comparison of microbial composition with the validation cohort. Data in panels G–I are supplemental to Fig. 3, A–C. Fig. S3 displays taxonomic composition at the phylum level and the absolute abundance of select taxa in irC and non-irC patients at distinct timepoints. Data in panels A–D are supplemental to Fig. 4, A and B, and data in panels E–H are supplemental to Fig. 4, D–G. Supplemental tables present clinical metadata, biosample accession numbers, summary statistics, and other information. Table S1 shows the patient clinical summary. Table S2 lists subject characteristics. Table S3 lists histology scores of irC severity in mice after colitis induction by TCT. Table S4 shows the total colitis severity score in mice after colitis induction by TCT averaged by microbiota donor. Table S5 shows metadata and metagenomic sequences of mice before and after irC induction by TCT. Table S6 shows MaAsLin2 bacteria analysis at pre-TCT or post-TCT timepoints in irC-associated microbiota-colonized mice. Table S7 lists the characteristics of healthy and Crohn’s subjects. Table S8 lists differential genera at irC-initial (first 10 days of active irC) versus pre-irC microbiome samples in irC patients using MaAsLin2 linear mixed effect model. Table S9 lists differential species at irC-initial (first 10 days of active irC) versus pre-irC microbiome samples in irC patients using MaAsLin2 linear mixed effect model. Table S10 shows sample accession numbers for human and mouse gut microbiota sequences.

## Supplementary Material

Table S1 shows the patient clinical summary.

Table S2 lists subject characteristics.

Table S3 lists histology scores of irColitis severity in mice after colitis induction by TCT.

Table S4 shows the total colitis severity score in mice after colitis induction by TCT averaged by microbiota donor.

Table S5 shows metadata and metagenomic sequences of mice before and after irC induction by TCT.

Table S6 shows MaAsLin2 bacteria analysis at pre-TCT or post-TCT timepoints in irC-associated microbiota-colonized mice.

Table S7 lists the characteristics of healthy and Crohn’s subjects.

Table S8 lists differential genus at irC-initial (first 10 days of active irC) versus pre-irC microbiome samples in irC patients using MaAsLin2 linear mixed effect model.

Table S9 lists differential species at irC-initial (first 10 days of active irC) versus pre-irColitis microbiome samples in irC patients using MaAsLin2 linear mixed effect model.

Table S10 sample accessions for human and mouse microbiota sequnces.

## Data Availability

The sequence data files (FASTQ) for all LC human and murine sequencing samples are stored in the Sequence Read Archive (SRA) under project number PRJNA1012329. Sequence data files for CVC metagenomic sequencing samples are available in the SRA under project number PRJNA961175 with specific irC-initial samples: SRR24288413, SRR24288446, SRR24288462, SRR24288437, SRR24288426. Sequences from healthy individuals and Crohn’s disease patients are available under accessions PRJNA637878 and PRJNA946744. The donor metadata and accession numbers used in this paper are also available in online supplemental materials.

## References

[bib1] Abu-Sbeih, H., and Y. Wang. 2020. Gut microbiome and immune checkpoint inhibitor-induced enterocolitis. Dig. Dis. Sci. 65:797–799. 10.1007/s10620-020-06103-x 32040664

[bib2] Aggarwala, V., I. Mogno, Z. Li, C. Yang, G.J. Britton, A. Chen-Liaw, J. Mitcham, G. Bongers, D. Gevers, J.C. Clemente, . 2021. Precise quantification of bacterial strains after fecal microbiota transplantation delineates long-term engraftment and explains outcomes. Nat. Microbiol. 6:1309–1318. 10.1038/s41564-021-00966-0 34580445 PMC8993687

[bib3] Ananthakrishnan, A.N., C. Luo, V. Yajnik, H. Khalili, J.J. Garber, B.W. Stevens, T. Cleland, and R.J. Xavier. 2017. Gut microbiome function predicts response to anti-integrin biologic therapy in Inflammatory Bowel diseases. Cell Host Microbe. 21:603–610.e3. 10.1016/j.chom.2017.04.010 28494241 PMC5705050

[bib4] Andrews, M.C., C.P.M. Duong, V. Gopalakrishnan, V. Iebba, W.S. Chen, L. Derosa, M.A.W. Khan, A.P. Cogdill, M.G. White, M.C. Wong, . 2021. Gut microbiota signatures are associated with toxicity to combined CTLA-4 and PD-1 blockade. Nat. Med. 27:1432–1441. 10.1038/s41591-021-01406-6 34239137 PMC11107795

[bib5] Arthur, J.C., E. Perez-Chanona, M. Mühlbauer, S. Tomkovich, J.M. Uronis, T.-J. Fan, B.J. Campbell, T. Abujamel, B. Dogan, A.B. Rogers, . 2012. Intestinal inflammation targets cancer-inducing activity of the microbiota. Science. 338:120–123. 10.1126/science.1224820 22903521 PMC3645302

[bib6] Atarashi, K., W. Suda, C. Luo, T. Kawaguchi, I. Motoo, S. Narushima, Y. Kiguchi, K. Yasuma, E. Watanabe, T. Tanoue, . 2017. Ectopic colonization of oral bacteria in the intestine drives T_H_1 cell induction and inflammation. Science. 358:359–365. 10.1126/science.aan4526 29051379 PMC5682622

[bib7] Baruch, E.N., I. Youngster, G. Ben-Betzalel, R. Ortenberg, A. Lahat, L. Katz, K. Adler, D. Dick-Necula, S. Raskin, N. Bloch, . 2021. Fecal microbiota transplant promotes response in immunotherapy-refractory melanoma patients. Science. 371:602–609. 10.1126/science.abb5920 33303685

[bib8] Britton, G.J., E.J. Contijoch, I. Mogno, O.H. Vennaro, S.R. Llewellyn, R. Ng, Z. Li, A. Mortha, M. Merad, A. Das, . 2019. Microbiotas from humans with inflammatory bowel disease alter the balance of gut Th17 and RORγt^+^ regulatory T cells and exacerbate colitis in mice. Immunity. 50:212–224.e4. 10.1016/j.immuni.2018.12.015 30650377 PMC6512335

[bib9] Britton, G.J., E.J. Contijoch, M.P. Spindler, V. Aggarwala, B. Dogan, G. Bongers, L. San Mateo, A. Baltus, A. Das, D. Gevers, . 2020. Defined microbiota transplant restores Th17/RORγt^+^ regulatory T cell balance in mice colonized with inflammatory bowel disease microbiotas. Proc. Natl. Acad. Sci. USA. 117:21536–21545. 10.1073/pnas.1922189117 32817490 PMC7474624

[bib10] Callahan, B.J., P.J. McMurdie, M.J. Rosen, A.W. Han, A.J.A. Johnson, and S.P. Holmes. 2016. DADA2: High-resolution sample inference from Illumina amplicon data. Nat. Methods. 13:581–583. 10.1038/nmeth.3869 27214047 PMC4927377

[bib11] Canales-Herrerias, P., Y. Garcia-Carmona, J. Shang, H. Meringer, D.S. Yee, L. Radigan, S. Buta, G. Martinez-Delgado, M. Tankelevich, D. Helmus, . 2023. Selective IgA2 deficiency in a patient with small intestinal Crohn’s disease. J. Clin. Invest. 133:e167742. 10.1172/JCI167742 37129981 PMC10266768

[bib12] Chaput, N., P. Lepage, C. Coutzac, E. Soularue, K. Le Roux, C. Monot, L. Boselli, E. Routier, L. Cassard, M. Collins, . 2017. Baseline gut microbiota predicts clinical response and colitis in metastatic melanoma patients treated with ipilimumab. Ann. Oncol. 28:1368–1379. 10.1093/annonc/mdx108 28368458

[bib13] Chassaing, B., G. Srinivasan, M.A. Delgado, A.N. Young, A.T. Gewirtz, and M. Vijay-Kumar. 2012. Fecal lipocalin 2, a sensitive and broadly dynamic non-invasive biomarker for intestinal inflammation. PLoS One. 7:e44328. 10.1371/journal.pone.0044328 22957064 PMC3434182

[bib14] Contijoch, E.J., G.J. Britton, C. Yang, I. Mogno, Z. Li, R. Ng, S.R. Llewellyn, S. Hira, C. Johnson, K.M. Rabinowitz, . 2019. Gut microbiota density influences host physiology and is shaped by host and microbial factors. Elife. 8:e40553. 10.7554/eLife.40553 30666957 PMC6342524

[bib15] Costello, S.P., P.A. Hughes, O. Waters, R.V. Bryant, A.D. Vincent, P. Blatchford, R. Katsikeros, J. Makanyanga, M.A. Campaniello, C. Mavrangelos, . 2019. Effect of fecal microbiota transplantation on 8-week remission in patients with ulcerative colitis: A randomized clinical trial. JAMA. 321:156–164. 10.1001/jama.2018.20046 30644982 PMC6439766

[bib16] Davar, D., A.K. Dzutsev, J.A. McCulloch, R.R. Rodrigues, J.M. Chauvin, R.M. Morrison, R.N. Deblasio, C. Menna, Q. Ding, O. Pagliano, . 2021. Fecal microbiota transplant overcomes resistance to anti-PD-1 therapy in melanoma patients. Science. 371:595–602. 10.1126/science.abf3363 33542131 PMC8097968

[bib17] Derosa, L., B. Routy, M. Fidelle, V. Iebba, L. Alla, E. Pasolli, N. Segata, A. Desnoyer, F. Pietrantonio, G. Ferrere, . 2020. Gut bacteria composition drives primary resistance to cancer immunotherapy in renal cell carcinoma patients. Eur. Urol. 78:195–206. 10.1016/j.eururo.2020.04.044 32376136

[bib18] Dubin, K., M.K. Callahan, B. Ren, R. Khanin, A. Viale, L. Ling, D. No, A. Gobourne, E. Littmann, C. Huttenhower, . 2016. Intestinal microbiome analyses identify melanoma patients at risk for checkpoint-blockade-induced colitis. Nat. Commun. 7:10391. 10.1038/ncomms10391 26837003 PMC4740747

[bib19] Elkrief, A., W. Nicholas, N. Smith, A. Dai, J.B. Slingerland, N. Aleynick, M.A. Lumish, P. Giardina, J.E. Chaft, P.B. Chapman, . 2023. Fecal microbiota transplantation for refractory immune-checkpoint-inhibitor colitis. J. Clin. Oncol. 41:2657. 10.1200/JCO.2023.41.16_suppl.2657

[bib20] Faith, J.J., J.L. Guruge, M. Charbonneau, S. Subramanian, H. Seedorf, A.L. Goodman, J.C. Clemente, R. Knight, A.C. Heath, R.L. Leibel, . 2013. The long-term stability of the human gut microbiota. Science. 341:1237439. 10.1126/science.1237439 23828941 PMC3791589

[bib21] Faith, J.J., N.P. McNulty, F.E. Rey, and J.I. Gordon. 2011. Predicting a human gut microbiota’s response to diet in gnotobiotic mice. Science. 333:101–104. 10.1126/science.1206025 21596954 PMC3303606

[bib22] Fidelle, M., C. Rauber, C. Alves Costa Silva, A.-L. Tian, I. Lahmar, A.M. de La Varende, L. Zhao, C. Thelemaque, I. Lebhar, M. Messaoudene, . 2023. A microbiota-modulated checkpoint directs immunosuppressive intestinal T cells into cancers. Science. 380:eabo2296. 10.1126/science.abo2296 37289890

[bib23] Frank, D.N., A.L. St Amand, R.A. Feldman, E.C. Boedeker, N. Harpaz, and N.R. Pace. 2007. Molecular-phylogenetic characterization of microbial community imbalances in human inflammatory bowel diseases. Proc. Natl. Acad. Sci. USA. 104:13780–13785. 10.1073/pnas.0706625104 17699621 PMC1959459

[bib24] Frankel, A.E., L.A. Coughlin, J. Kim, T.W. Froehlich, Y. Xie, E.P. Frenkel, and A.Y. Koh. 2017. Metagenomic shotgun sequencing and unbiased metabolomic profiling identify specific human gut microbiota and metabolites associated with immune checkpoint therapy efficacy in melanoma patients. Neoplasia. 19:848–855. 10.1016/j.neo.2017.08.004 28923537 PMC5602478

[bib25] Gao, Y., P. Xu, D. Sun, Y. Jiang, X.L. Lin, T. Han, J. Yu, C. Sheng, H. Chen, J. Hong, . 2023. Faecalibacterium prausnitzii abrogates intestinal toxicity and promotes tumor immunity to increase the efficacy of dual CTLA-4 and PD-1 checkpoint blockade. Cancer Res. 83:3710–3725. 10.1158/0008-5472.CAN-23-0605 37602831

[bib26] Gloor, G.B., J.M. Macklaim, V. Pawlowsky-Glahn, and J.J. Egozcue. 2017. Microbiome datasets are compositional: And this is not optional. Front. Microbiol. 8:2224. 10.3389/fmicb.2017.02224 29187837 PMC5695134

[bib27] Haifer, C., S. Paramsothy, N.O. Kaakoush, A. Saikal, S. Ghaly, T. Yang, L.D.W. Luu, T.J. Borody, and R.W. Leong. 2022. Lyophilised oral faecal microbiota transplantation for ulcerative colitis (LOTUS): A randomised, double-blind, placebo-controlled trial. Lancet Gastroenterol. Hepatol. 7:141–151. 10.1016/S2468-1253(21)00400-3 34863330

[bib28] Halsey, T.M., A.S. Thomas, T. Hayase, W. Ma, H. Abu-Sbeih, B. Sun, E.R. Parra, Z.-D. Jiang, H.L. DuPont, C. Sanchez, . 2023. Microbiome alteration via fecal microbiota transplantation is effective for refractory immune checkpoint inhibitor-induced colitis. Sci. Transl. Med. 15:eabq4006. 10.1126/scitranslmed.abq4006 37315113 PMC10759507

[bib29] Heul, A.V., and T. Stappenbeck. 2018. Establishing A mouse model of checkpoint inhibitor-induced colitis: Pilot experiments and future directions. J. Allergy Clin. Immunol. 141:AB119. 10.1016/j.jaci.2017.12.378

[bib30] Hooper, L.V., and A.J. Macpherson. 2010. Immune adaptations that maintain homeostasis with the intestinal microbiota. Nat. Rev. Immunol. 10:159–169. 10.1038/nri2710 20182457

[bib31] Khoja, L., D. Day, T. Wei-Wu Chen, L.L. Siu, and A.R. Hansen. 2017. Tumour- and class-specific patterns of immune-related adverse events of immune checkpoint inhibitors: A systematic review. Ann. Oncol. 28:2377–2385. 10.1093/annonc/mdx286 28945858

[bib32] Koelink, P.J., M.E. Wildenberg, L.W. Stitt, B.G. Feagan, M. Koldijk, A.B. van ’t Wout, R. Atreya, M. Vieth, J.F. Brandse, S. Duijst, . 2018. Development of reliable, valid and responsive scoring systems for endoscopy and histology in animal models for inflammatory bowel disease. J. Crohns Colitis. 12:794–803. 10.1093/ecco-jcc/jjy035 29608662 PMC6022651

[bib33] Lee, K.A., A.M. Thomas, L.A. Bolte, J.R. Björk, L.K. de Ruijter, F. Armanini, F. Asnicar, A. Blanco-Miguez, R. Board, N. Calbet-Llopart, . 2022. Cross-cohort gut microbiome associations with immune checkpoint inhibitor response in advanced melanoma. Nat. Med. 28:535–544. 10.1038/s41591-022-01695-5 35228751 PMC8938272

[bib34] Liu, T., Q. Xiong, L. Li, and Y. Hu. 2019. Intestinal microbiota predicts lung cancer patients at risk of immune-related diarrhea. Immunotherapy. 11:385–396. 10.2217/imt-2018-0144 30693820

[bib35] Llewellyn, S.R., G.J. Britton, E.J. Contijoch, O.H. Vennaro, A. Mortha, J.-F. Colombel, A. Grinspan, J.C. Clemente, M. Merad, and J.J. Faith. 2018. Interactions between diet and the intestinal microbiota alter intestinal permeability and colitis severity in mice. Gastroenterology. 154:1037–1046.e2. 10.1053/j.gastro.2017.11.030 29174952 PMC5847454

[bib36] Lo, J.W., D. Cozzetto, J.L. Alexander, N.P. Danckert, M. Madgwick, N. Knox, J.Y.X. Sieh, M. Olbei, Z. Liu, H. Ibraheim, . 2023. Immune checkpoint inhibitor-induced colitis is mediated by polyfunctional lymphocytes and is dependent on an IL23/IFNγ axis. Nat. Commun. 14:6719. 10.1038/s41467-023-41798-2 37872166 PMC10593820

[bib37] Machiels, K., M. Joossens, J. Sabino, V. De Preter, I. Arijs, V. Eeckhaut, V. Ballet, K. Claes, F. Van Immerseel, K. Verbeke, . 2014. A decrease of the butyrate-producing species Roseburia hominis and Faecalibacterium prausnitzii defines dysbiosis in patients with ulcerative colitis. Gut. 63:1275–1283. 10.1136/gutjnl-2013-304833 24021287

[bib38] Maghini, D.G., M. Dvorak, A. Dahlen, M. Roos, S. Kuersten, and A.S. Bhatt. 2023. Quantifying bias introduced by sample collection in relative and absolute microbiome measurements. Nat. Biotechnol. 42:328–338. 10.1038/s41587-023-01754-3 37106038

[bib39] Matson, V., J. Fessler, R. Bao, T. Chongsuwat, Y. Zha, M.L. Alegre, J.J. Luke, and T.F. Gajewski. 2018. The commensal microbiome is associated with anti-PD-1 efficacy in metastatic melanoma patients. Science. 359:104–108. 10.1126/science.aao3290 29302014 PMC6707353

[bib40] McCulloch, J.A., D. Davar, R.R. Rodrigues, J.H. Badger, J.R. Fang, A.M. Cole, A.K. Balaji, M. Vetizou, S.M. Prescott, M.R. Fernandes, . 2022. Intestinal microbiota signatures of clinical response and immune-related adverse events in melanoma patients treated with anti-PD-1. Nat. Med. 28:545–556. 10.1038/s41591-022-01698-2 35228752 PMC10246505

[bib41] McMurdie, P.J., and S. Holmes. 2013. phyloseq: an R package for reproducible interactive analysis and graphics of microbiome census data. PLoS One. 8:e61217. 10.1371/journal.pone.0061217 23630581 PMC3632530

[bib42] Moayyedi, P., M.G. Surette, P.T. Kim, J. Libertucci, M. Wolfe, C. Onischi, D. Armstrong, J.K. Marshall, Z. Kassam, W. Reinisch, and C.H. Lee. 2015. Fecal microbiota transplantation induces remission in patients with active ulcerative colitis in a randomized controlled trial. Gastroenterology. 149:102–109.e6. 10.1053/j.gastro.2015.04.001 25857665

[bib43] Oksanen, J., G.L. Simpson, G.F. Blanchet, P. Legendre, P.R. Minchin, R.B. O’Hara, P. Solymos, H.H. Stevens, E. Szoecs, H. Wagner, . 2023. vegan: Community ecology package.

[bib44] Paramsothy, S., M.A. Kamm, N.O. Kaakoush, A.J. Walsh, J. van den Bogaerde, D. Samuel, R.W.L. Leong, S. Connor, W. Ng, R. Paramsothy, . 2017. Multidonor intensive faecal microbiota transplantation for active ulcerative colitis: A randomised placebo-controlled trial. Lancet. 389:1218–1228. 10.1016/S0140-6736(17)30182-4 28214091

[bib45] Powrie, F., M.W. Leach, S. Mauze, L.B. Caddle, and R.L. Coffman. 1993. Phenotypically distinct subsets of CD4+ T cells induce or protect from chronic intestinal inflammation in C. B-17 scid mice. Int. Immunol. 5:1461–1471. 10.1093/intimm/5.11.1461 7903159

[bib46] Quast, C., E. Pruesse, P. Yilmaz, J. Gerken, T. Schweer, P. Yarza, J. Peplies, and F.O. Glöckner. 2013. The SILVA ribosomal RNA gene database project: Improved data processing and web-based tools. Nucleic Acids Res. 41:D590–D596. 10.1093/nar/gks1219 23193283 PMC3531112

[bib47] Raygoza Garay, J.A., W. Turpin, S.-H. Lee, M.I. Smith, A. Goethel, A.M. Griffiths, P. Moayyedi, O. Espin-Garcia, M. Abreu, G.L. Aumais, . 2023. Gut microbiome composition is associated with future onset of Crohn’s disease in healthy first-degree relatives. Gastroenterology. 165:670–681. 10.1053/j.gastro.2023.05.032 37263307

[bib48] Rojas-Tapias, D.F., E.M. Brown, E.R. Temple, M.A. Onyekaba, A.M.T. Mohamed, K. Duncan, M. Schirmer, R.L. Walker, T. Mayassi, K.A. Pierce, . 2022. Inflammation-associated nitrate facilitates ectopic colonization of oral bacterium Veillonella parvula in the intestine. Nat. Microbiol. 7:1673–1685. 10.1038/s41564-022-01224-7 36138166 PMC9728153

[bib49] Rossen, N.G., J.K. MacDonald, E.M. de Vries, G.R. D’Haens, W.M. de Vos, E.G. Zoetendal, and C.Y. Ponsioen. 2015. Fecal microbiota transplantation as novel therapy in gastroenterology: A systematic review. World J. Gastroenterol. 21:5359–5371. 10.3748/wjg.v21.i17.5359 25954111 PMC4419078

[bib50] Routy, B., E. Le Chatelier, L. Derosa, C.P.M. Duong, M.T. Alou, R. Daillère, A. Fluckiger, M. Messaoudene, C. Rauber, M.P. Roberti, . 2018. Gut microbiome influences efficacy of PD-1-based immunotherapy against epithelial tumors. Science. 359:91–97. 10.1126/science.aan3706 29097494

[bib51] Routy, B., J.G. Lenehan, W.H. Miller Jr., R. Jamal, M. Messaoudene, B.A. Daisley, C. Hes, K.F. Al, L. Martinez-Gili, M. Punčochář, . 2023. Fecal microbiota transplantation plus anti-PD-1 immunotherapy in advanced melanoma: a phase I trial. Nat. Med. 29:2121–2132. 10.1038/s41591-023-02453-x 37414899

[bib52] RStudio Team . 2020. RStudio: Integrated development for R.

[bib53] Schirmer, M., L. Denson, H. Vlamakis, E.A. Franzosa, S. Thomas, N.M. Gotman, P. Rufo, S.S. Baker, C. Sauer, J. Markowitz, . 2018. Compositional and temporal changes in the gut microbiome of pediatric ulcerative colitis patients are linked to disease course. Cell Host Microbe. 24:600–610.e4. 10.1016/j.chom.2018.09.009 30308161 PMC6277984

[bib54] Segata, N., J. Izard, L. Waldron, D. Gevers, L. Miropolsky, W.S. Garrett, and C. Huttenhower. 2011. Metagenomic biomarker discovery and explanation. Genome Biol. 12:R60. 10.1186/gb-2011-12-6-r60 21702898 PMC3218848

[bib55] Shaikh, F.Y., J.R. White, J.J. Gills, T. Hakozaki, C. Richard, B. Routy, Y. Okuma, M. Usyk, A. Pandey, J.S. Weber, . 2021. A Uniform computational approach improved on existing pipelines to reveal microbiome biomarkers of nonresponse to immune checkpoint inhibitors. Clin. Cancer Res. 27:2571–2583. 10.1158/1078-0432.CCR-20-4834 33593881 PMC9053858

[bib56] Shannon, C.E. 1948. A mathematical theory of communication. Bell Syst. Tech. J. 27:379–423. 10.1002/j.1538-7305.1948.tb01338.x

[bib57] Shin, N.-R., T.W. Whon, and J.-W. Bae. 2015. Proteobacteria: Microbial signature of dysbiosis in gut microbiota. Trends Biotechnol. 33:496–503. 10.1016/j.tibtech.2015.06.011 26210164

[bib58] Simpson, R.C., E.R. Shanahan, M. Batten, I.L.M. Reijers, M. Read, I.P. Silva, J.M. Versluis, R. Ribeiro, A.S. Angelatos, J. Tan, . 2022. Diet-driven microbial ecology underpins associations between cancer immunotherapy outcomes and the gut microbiome. Nat. Med. 28:2344–2352. 10.1038/s41591-022-01965-2 36138151

[bib59] Sivan, A., L. Corrales, N. Hubert, J.B. Williams, K. Aquino-Michaels, Z.M. Earley, F.W. Benyamin, Y.M. Lei, B. Jabri, M.L. Alegre, . 2015. Commensal Bifidobacterium promotes antitumor immunity and facilitates anti-PD-L1 efficacy. Science. 350:1084–1089. 10.1126/science.aac4255 26541606 PMC4873287

[bib60] Sokol, H., P. Seksik, J.P. Furet, O. Firmesse, I. Nion-Larmurier, L. Beaugerie, J. Cosnes, G. Corthier, P. Marteau, and J. Doré. 2009. Low counts of Faecalibacterium prausnitzii in colitis microbiota. Inflamm. Bowel Dis. 15:1183–1189. 10.1002/ibd.20903 19235886

[bib61] Som, A., R. Mandaliya, D. Alsaadi, M. Farshidpour, A. Charabaty, N. Malhotra, and M.C. Mattar. 2019. Immune checkpoint inhibitor-induced colitis: A comprehensive review. World J. Clin. Cases. 7:405–418. 10.12998/wjcc.v7.i4.405 30842952 PMC6397821

[bib62] Spencer, C.N., J.L. McQuade, V. Gopalakrishnan, J.A. McCulloch, M. Vetizou, A.P. Cogdill, M.A.W. Khan, X. Zhang, M.G. White, C.B. Peterson, . 2021. Dietary fiber and probiotics influence the gut microbiome and melanoma immunotherapy response. Science. 374:1632–1640. 10.1126/science.aaz7015 34941392 PMC8970537

[bib63] Stewart, C.J., N.J. Ajami, J.L. O’Brien, D.S. Hutchinson, D.P. Smith, M.C. Wong, M.C. Ross, R.E. Lloyd, H. Doddapaneni, G.A. Metcalf, . 2018. Temporal development of the gut microbiome in early childhood from the TEDDY study. Nature. 562:583–588. 10.1038/s41586-018-0617-x 30356187 PMC6415775

[bib64] Tiwana, H., R.S. Natt, R. Benitez-Brito, S. Shah, C. Wilson, S. Bridger, M. Harbord, M. Sarner, and A. Ebringer. 2001. Correlation between the immune responses to collagens type I, III, IV and V and Klebsiella pneumoniae in patients with Crohn’s disease and ankylosing spondylitis. Rheumatology. 40:15–23. 10.1093/rheumatology/40.1.15 11157137

[bib65] Usyk, M., A. Pandey, R.B. Hayes, U. Moran, A. Pavlick, I. Osman, J.S. Weber, and J. Ahn. 2021. Bacteroides vulgatus and Bacteroides dorei predict immune-related adverse events in immune checkpoint blockade treatment of metastatic melanoma. Genome Med. 13:160. 10.1186/s13073-021-00974-z 34641962 PMC8513370

[bib66] Vandeputte, D., L. De Commer, R.Y. Tito, G. Kathagen, J. Sabino, S. Vermeire, K. Faust, and J. Raes. 2021. Temporal variability in quantitative human gut microbiome profiles and implications for clinical research. Nat. Commun. 12:6740. 10.1038/s41467-021-27098-7 34795283 PMC8602282

[bib67] Vétizou, M., J.M. Pitt, R. Daillère, P. Lepage, N. Waldschmitt, C. Flament, S. Rusakiewicz, B. Routy, M.P. Roberti, C.P.M. Duong, . 2015. Anticancer immunotherapy by CTLA-4 blockade relies on the gut microbiota. Science. 350:1079–1084. 10.1126/science.aad1329 26541610 PMC4721659

[bib68] Wang, F., Q. Yin, L. Chen, and M.M. Davis. 2018a. Bifidobacterium can mitigate intestinal immunopathology in the context of CTLA-4 blockade. Proc. Natl. Acad. Sci. USA. 115:157–161. 10.1073/pnas.1712901115 29255057 PMC5776803

[bib69] Wang, T., N. Zheng, Q. Luo, L. Jiang, B. He, X. Yuan, and L. Shen. 2019. Probiotics Lactobacillus reuteri abrogates immune checkpoint blockade-associated colitis by inhibiting group 3 innate lymphoid cells. Front. Immunol. 10:1235. 10.3389/ffimmu.2019.01235 31214189 PMC6558076

[bib70] Wang, Y., W. Ma, H. Abu-Sbeih, Z.-D. Jiang, and H.L. DuPont. 2020. Fecal microbiota transplantation (FMT) for immune checkpoint inhibitor induced–colitis (IMC) refractory to immunosuppressive therapy. J. Clin. Oncol. 38:3067. 10.1200/JCO.2020.38.15_suppl.3067

[bib71] Wang, Y., D.H. Wiesnoski, B.A. Helmink, V. Gopalakrishnan, K. Choi, H.L. DuPont, Z.D. Jiang, H. Abu-Sbeih, C.A. Sanchez, C.C. Chang, . 2018b. Fecal microbiota transplantation for refractory immune checkpoint inhibitor-associated colitis. Nat. Med. 24:1804–1808. 10.1038/s41591-018-0238-9 30420754 PMC6322556

[bib72] Winter, S.E., M.G. Winter, M.N. Xavier, P. Thiennimitr, V. Poon, A.M. Keestra, R.C. Laughlin, G. Gomez, J. Wu, S.D. Lawhon, . 2013. Host-derived nitrate boosts growth of E. coli in the inflamed gut. Science. 339:708–711. 10.1126/science.1232467 23393266 PMC4004111

[bib73] Yatsunenko, T., F.E. Rey, M.J. Manary, I. Trehan, M.G. Dominguez-Bello, M. Contreras, M. Magris, G. Hidalgo, R.N. Baldassano, A.P. Anokhin, . 2012. Human gut microbiome viewed across age and geography. Nature. 486:222–227. 10.1038/nature11053 22699611 PMC3376388

[bib74] Zhou, Y., Y.B. Medik, B. Patel, D.B. Zamler, S. Chen, T. Chapman, S. Schneider, E.M. Park, R.L. Babcock, T.T. Chrisikos, . 2023. Intestinal toxicity to CTLA-4 blockade driven by IL-6 and myeloid infiltration. J. Exp. Med. 220:e20221333. 10.1084/jem.20221333 36367776 PMC9664499

